# Everything everywhere all at once? Disentangling the long-lasting riddle of phylogenetic relationships and cryptic hybridization in the amphitropical genus *Larrea*

**DOI:** 10.1093/aobpla/plaf024

**Published:** 2025-04-25

**Authors:** María Paula Quiroga, Lucia V Castello, Mariana Tadey, Sebastián Márquez, Andrea C Premoli, Cintia P Souto

**Affiliations:** Universidad Nacional del Comahue, Centro Regional Universitario Bariloche-INIBIOMA-CONICET, Pasaje Gutiérrez 1514, Bariloche, Argentina; Departamento de Botánica, Universidad Nacional del Comahue, Quintral 1250, CP 8400, Bariloche, Río Negro, Argentina; Universidad Nacional del Comahue, Centro Regional Universitario Bariloche-INIBIOMA-CONICET, Pasaje Gutiérrez 1514, Bariloche, Argentina; Universidad Nacional del Comahue, Centro Regional Universitario Bariloche-INIBIOMA-CONICET, Pasaje Gutiérrez 1514, Bariloche, Argentina; Universidad Nacional del Comahue, Centro Regional Universitario Bariloche-INIBIOMA-CONICET, Pasaje Gutiérrez 1514, Bariloche, Argentina; Universidad Nacional del Comahue, Centro Regional Universitario Bariloche-INIBIOMA-CONICET, Pasaje Gutiérrez 1514, Bariloche, Argentina; Universidad Nacional del Comahue, Centro Regional Universitario Bariloche-INIBIOMA-CONICET, Pasaje Gutiérrez 1514, Bariloche, Argentina

**Keywords:** chloroplast sequences, creosote bush, cryptic hybridization, hybrid swarm, Monte Desert, North and South American drylands, nuclear sequences

## Abstract

The genus *Larrea* has an amphitropical distribution in North and South American deserts, and its phylogeny remains unresolved. This genus is conspicuous and specious within the Monte Desert, the largest, although understudied, southern South American dryland. *Larrea* presents an interesting case for phylogenetic studies due to its paternally inherited chloroplasts, its species hybridize in nature, and although nominal species are morphologically distinct, hybrids might be cryptic. We analysed ITS2 nuclear (nDNA) and rbcL chloroplast (cpDNA) sequences of the bifoliolate section, *Bifolium*, including *L. tridentata* (*Lt*) from North America, and its South American congeners: *L. cuneifolia* (*Lc*) and *L. divaricata* (*Ld*), and sequences of the multifoliolate *Larrea* section: *L. ameghinoi* (*La*), *L. nitida* (*Ln*), and a morphological hybrid swarm. We aligned and analysed sequences from 111 individuals collected at 31 populations sampled along the range of each species. The nDNA revealed 56 haplotypes, and median-joining and maximum likelihood reconstructions provided clear separation among species and suggested hybridization between *Lc*-*Ld*. The nuclear phylogeny showed that the section *Larrea* diverged earlier than *Bifolium,* within which *Lc* diverged first, meanwhile, consistent with previous studies, *Lt* forms a monophyletic group sister to *Ld*. Comparatively, cpDNA was less variable, with only six haplotypes shared between *Ln-Lc* and *Ln*-*La*, and rarely between *Ld*-*Lc*. Our results emphasize the significance of separately considering nuclear and plastid evolutionary signals when reconstructing unresolved relationships. While nuclear markers clarified phylogenetic relationships and cryptic hybridization among *Larrea* species, the chloroplast revealed the retention of widespread ancient polymorphisms, which were conserved in populations of distinct species. Each marker provided insights into particular evolutionary patterns, highlighting that genetic variation may be more influenced by hybridization and mode of chloroplast inheritance than previously recognized.

## Introduction

Drylands cover nearly 41% of Earth’s land surface, and its flora is worldwide highly threatened by environmental degradation. In particular, the Monte Desert is the largest southern South American dryland with at least 30% of endemic biota, considerably understudied (Roig et al. 2009). This region has been proposed as a ‘Hotspot’, as many genera occur with amphitropical distribution in the North American and South American deserts (Lia et al. 2001; Moore et al. 2012; Amarilla et al. 2015; Simpson et al. 2017a, b; Quiroga et al. 2018). Furthermore, dryland vegetation has evolved in the temperate Neotropics since the Oligocene (Burnham and Graham 1999), encompassing heterogeneous climates and topography. The Zygophyllaceae family is a key component of dryland floras worldwide, with the genus *Larrea* consistently dominating its amphitropical range in North and South American deserts. Four species are found in Argentina’s Monte Desert while a single species prevails in North America (Hunter 1995; Roig et al. 2009). Despite many attempts to solve the phylogenetic relationships among its species, the reconstruction of the evolutionary history of this genus is still an enigma (Hunter 1995; Lia et al. 2001; Laport et al. 2012).

In the past, phylogenetic affinities within the genus have been inferred based on morphological, cytogenetic, biochemical, and molecular studies (Hunziker et al. 1977; Yang et al. 1977; Hunziker 1978; Lia et al. 2001; Laport et al. 2012). The molecular phylogenetic relationships within the genus *Larrea* have been interpreted as the result of hybridization. However, limited species’ sampling and incongruent phylogenetic patterns between chloroplast and nuclear markers (Lia et al. 2001; Laport et al. 2012) may have biased such reconstructions (See Supplementary Table S1).

Gene flow in *Larrea* species is spatially restricted by pollen and seed dispersal. Pollination is usually mediated by small solitary bees with localized territorial behaviour (Tadey 2015). Meanwhile, it is common to see accumulated seeds below maternal plants and neighbours, although, its schizocarp fruits might have epizoic dispersal, transported by mammals or birds (Hunziker et al. 1977). As gene flow in plants is affected by pollen and seed dispersal, it is important to consider the type of inheritance of markers to be used in phylogenetic reconstructions. To avoid such inconsistencies, separately analysed nuclear and chloroplast DNA sequences using comprehensive sampling methods are needed (Rieseberg and Soltis 1991; Soltis et al. 2007, 2010).

Hybridization followed by asymmetric backcrossing, i.e. introgression, is an important source of evolutionary novelties and can promote speciation, and frequently, polyploidization (Soltis et al. 2015; Stull et al. 2023). Moreover, shared ancestral variation can be evidenced when a polymorphism occurs in two sister species (Li et al. 2022). Among closely related angiosperms, extensive exchange of chloroplast DNA (cpDNA) haplotypes is commonly observed, possibly due to introgression as well as shared ancestral polymorphism (Acosta and Premoli 2010; Li et al. 2022). In most angiosperms, cpDNA is maternally inherited only, dispersed through seeds and thus has less potential for intraspecific gene flow (Petit et al. 2005), which hinders intraspecific homogenization. However, in plants with paternally inherited cpDNA via pollen, such as in *Larrea* and a few other angiosperms (Yang et al. 2000), this could facilitate plastid homogenization, as occurs in gymnosperms (Mogensen 1996). In addition, the low mutation rate of cpDNA may also slow lineage sorting and result in extensive exchange of ancestral haplotypes between sister species (Zhou et al. 2010). Particularly if this occurs in a shared geographic origin (ancestral sympatry), sister species would be expected to share plastidial haplotypic variants (Li et al. 2022).

In addition, cpDNA retention of ancestral polymorphisms might produce similar patterns of shared genetic diversity (Zhou et al. 2017), which would be difficult to unravel since they might be found in distant populations. In contrast, secondary gene flow and introgression, most probably among neighbouring populations, would result in lower levels of interspecific genetic differentiation than between distant populations (Petit and Excoffier 2009). Therefore, shared cpDNA haplotypes in sympatry and/or parapatry could result from hybridization followed by introgression, whereas shared haplotypes in allopatry may indicate the retention of ancestral polymorphisms. Under these hypotheses, the spatial, i.e. geographic, dimension as well as the temporal one, i.e. dating methods, of intra- and interspecific variation becomes relevant in phylogenetic studies.

The genus *Larrea* is polymorphic in terms of ploidy levels. The only species inhabiting North American deserts, *L. tridentata* (DC) Coville, comprises diploid, tetraploid, and hexaploid populations that interdigitate geographically. Previous phylogeographic studies suggested that polyploidy originated during the establishment of *L. tridentata* along its rapid expansion, and the different cytotypes are considered a single species (Yang 1970; Hunziker et al. 1972; Laport et al. 2012; Laport and Ramsey 2015; Vidal-Russell et al. 2022). In southern South America, the genus *Larrea* consists of three diploid species *L. nitida* Cav.*, L. ameghinoi* Speg*, L. divaricata* Cav. and one tetraploid *L. cuneifolia* Cav. whose hybrid origin between *L. nitida* and *L. divaricata* has been proposed (Lia et al. 2001; Vidal-Russell et al. 2022). Thus *L. nitida* would have acted as the chloroplast donor, and *L. divaricata* has been suggested as one of the nuclear donors based on anatomical, morphological, and cytogenetic evidence (Hunziker et al. 1977; Lia et al. 2001). Meanwhile, dot-blot hybridization by means of cytogenetic methods showed that *L. cuneifolia* has a nuclear genome largely homologous to *L. divaricata* (Poggio et al. 1989). However, the internal transcribed spacer (ITS) phylogeny supported a monophyletic clade for *L. nitida*, *L. ameghinoi*, and *L. cuneifolia* (Lia et al. 2001). On the other hand, Hunter (1995) obtained double-stranded DNA from the ITS region of single *Larrea* individuals, identifying more than one sequence for the same individual through cloning. These putative sequences typically matched with those of a different *Larrea* species. When building the phylogeny, Hunter (1995) found that *L. divaricata*/*L. tridentata* complex was ancestral to *L. nitida*, and *L. ameghinoi* was ancestral to the rest of the genus. These results contradicted the morphological systematics that grouped *L. nitida* and *L. ameghinoi* in the multifoliate *Larrea* section, which is ancestral to the rest of the genus. All these studies left unsolved phylogenetic relationships within the genus *Larrea*.

Barcoding in plants uses specific regions of chloroplast and nuclear DNA as a tool to identify species (Hebert et al. 2003), in a manner that a reduced number of markers might effectively track plant species boundaries (Fazekas et al. 2012). Barcode regions have many strengths: they are conserved, easily amplifiable, and helpful in identifying closely related species (Letsiou et al. 2024). Yet molecular markers with distinct modes of inheritance, i.e. nuclear biparental and chloroplast uniparental, could reflect conflicting phylogenetic signals (Yang et al. 2000). These markers have been effectively used for rapid species identification and phylogenetic analysis (de Vere et al. 2015; Batley 2016; Emerson 2025). The ITS2 and rbcL gene have demonstrated their utility in taxonomic classification and phylogenetic reconstruction at both the genus and species levels (Yao et al. 2010; Hollingsworth et al. 2011). The significance of selecting a gene for species delimitation lies in its ability to test hypotheses of hybridization and/or chloroplast capture, meanwhile, more variable or less conserved regions may introduce redundancies that are less informative. The use of a low number of markers in phylogenetic reconstructions may seem limited compared to other molecular techniques (e.g. single-nucleotide polymorphisms or SNPs), however, they offer valuable insights, particularly for a phytogeographic region that has been understudied and for a genus with unresolved interspecific relationships (Kartzinel et al. 2025) as *Larrea*. These barcode markers provide a cost-effective approach, enabling a comprehensive analysis of DNA sequences across multiple individuals, species, and localities, a data set not commonly found in the literature for this genus.

The unique life history traits of the genus *Larrea* provide an interesting and complex opportunity for studying its phylogeny. In this manuscript, we propose using nuclear spacer (ITS2) and rbcL chloroplast gene sequences to resolve phylogenetic relationships within this amphitropical genus, and to estimate divergence times among its species. To achieve this, we analysed barcode sequences from all five species across their native ranges, including several localities in the South American Monte Desert in Argentina for *L. ameghinoi*, *L. cuneifolia, L. divaricata*, and *L. nitida*, as well as a few North American desert localities for *L. tridentata*.

## Materials and methods

### Study system and sample collection

**Figure 1.**
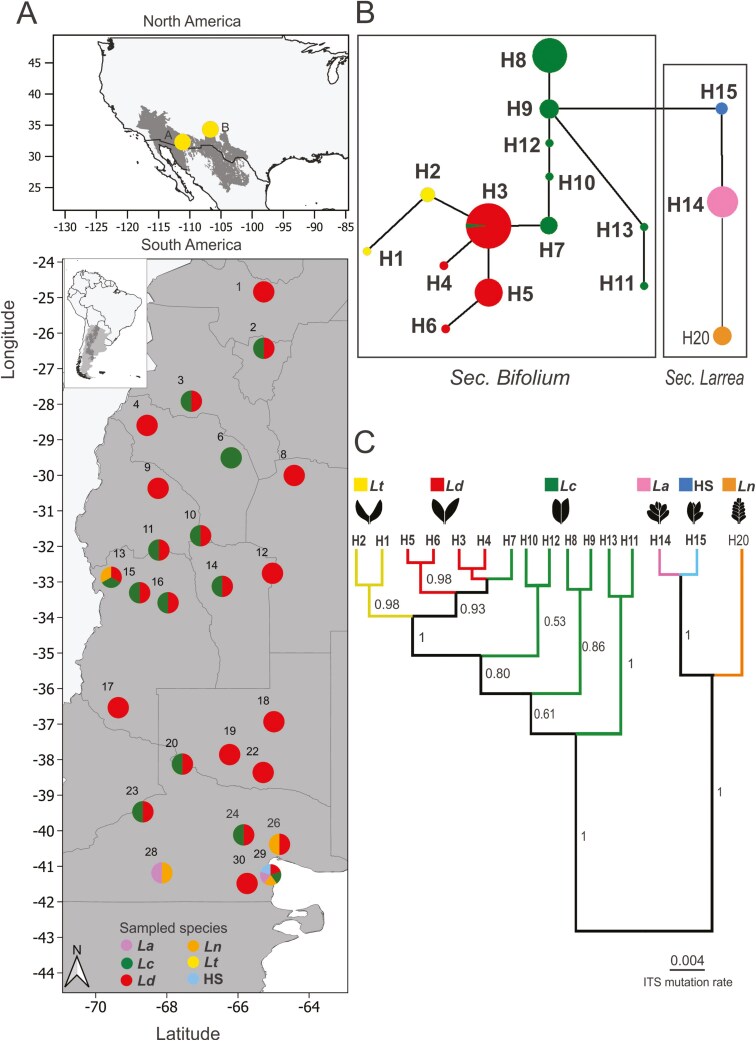
Sampled locations, ITS pure haplotypes network, and phylogenetic tree showing relationships among *Larrea* species. (A) Maps of sampled locations of *Larrea* species in North and South America, including population reference as in Table 1, with circle colours depicting sampled species at each location. (B) ITS pure haplotypes network, squares group sections *Bifolium* and *Larrea*. Circle sizes are proportional to haplotype frequencies, and between-haplotype branch length is proportional to the number of mutational changes. (C) ITS pure haplotypes phylogeny with the numbers on the right of the branches indicating Bayesian posterior probabilities. Colour codes and haplotype numbers: *Lt* in yellow (H1 and H2), *La* in pink (H14), *Lc* in green (H7–H13), *Ld* in red (H3–H6), *Ln* in orange (H20), HS in cyan (H15).

We studied the five species of the genus *Larrea* (Hunziker et al. 1972) (Table 1; Fig. 1A). South American species included the extremely rare *L*. *ameghinoi* (*N* = 9), and the more widespread and abundant *L. cuneifolia* (*N* = 26), *L*. *divaricata* (*N* = 57), and *L*. *nitida* (N = 9), hereafter referred as *La*, *Lc*, *Ld*, and *Ln*, respectively. We also sampled morpho hybrids (*N* = 6), previously cited as a hybrid swarm individual, hereafter HS, resulting from hybridization between different species in the unique location (RN-RN3), where these individuals co-occur with all South American species (Hunziker et al. 1969). These species were sampled in 31 localities, where one or more of the focal species can co-occur, covering the wide range of environments inhabited by each species, collecting individuals morphologically identified as ‘nominal species’ to differentiate between actual species and hybrids. We sampled across their respective ranges in the Monte Desert, an extensive phytogeographic region in central-western Argentina, spanning from 25° to 45° South latitude. The fifth species included in this study is *L*. *tridentata*, from now on *Lt,* (Yang 1970; Hunziker et al. 1972), which is endemic to North American deserts, and occurs between 21° and 36° North latitude. Despite the widespread range of *Lt* in North America, and due to budgetary limitations, we only sampled two localities and collected four individuals (*N* = 4). There are two sections within the genus *Larrea*, these evergreen xerophytic shrubs are easily identifiable mainly by their leaf morphology, number, and percentage of coalescence of leaflets, presence or absence of mucrones and rachis, and the shape of the stipules but also by fruit hairiness: the multifoliolate section *Larrea* includes *Ln* (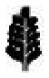) and *La* (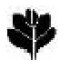), and the bifoliolate section, *Bifolium* includes *Lc* (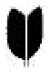), *Ld* (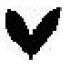) and *Lt* (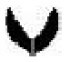) (Hunziker et al. 1977). All specimens were deposited in the Herbarium BCRU, Universidad Nacional del Comahue, at Centro Regional Universitario Bariloche, Argentina.

**Table 1.** Information on *Larrea* species sampled localities from North and South America. Including observed haplotypes at the nuclear ITS region -with the prefix H-, and in brackets chloroplast gene rbcL haplotypes -with the prefix cpH-. Also reporting the number of individuals sequenced per species at each locality. In **bold** pure haplotypes, lighter colors double-stranded DNA sequences of ITS, i.e. putative haplotypes PH1; and darker colors and *italics* PH2. Interspecific variants are represented with *IV*. Unique haplotypes are underlined. Highlighted in grey are indicated pure haplotypes from one species in an individual morphologically identified in the field as a different species.

Population reference	Population acronym	Latitude	Longitude	*Lt , La , Lc , Ld , Ln ,* HS Haplotypes	Sequenced individuals ITS and (rbcL)
A	NM-SC	34.324	−106.706	**H1**, **H2**, H27, H28 ( cpH 1)	2 *Lt,* (2 *Lt*)
B	TC-SNP	32.296	−111.166	**H2**, H27, H28 ( cpH 1)	2 *Lt,* (2 *Lt*)
1	SA-LC	−25.139	−65.575	**H3**, **H5**(cpH2)	4 *Ld,* (3 *Ld*)
2	SA-CA	−26.202	−65.573	**H10**, **H3**(cpH4) ; **H3, H4,** H17, *H29 IV, H30* (cpH2)	1 *Lc,* (2 *Lc*); 4 *Ld* (4 *Ld*)
3	CA-FBL	−27.690	−67.610	**H3,** H16, *H31 IV, H32, H33 IV*	3 *Ld*
4	LR-LB	−28.371	−68.855	**H5**	3 *Ld*
5	LR-FA	−29.000	−67.550	(cpH4)	(1 *Lc*)
6	LR-LA	−29.283	−66.491	**H7**, **H9**, **H10**, **H13**(cpH4)	3 *Lc,* (3 *Lc*)
7	SJ-SG	−29.497	−69.199	(cpH2)	(2 *Lc*)
8	CD-LBM	−29.780	−64.718	**H3, H5**, H16, H18, *H55*, *H56* ( cpH 2)	4 *Ld,* (3 *Ld*)
9	SJ-LC	−30.142	−68.545	**H3**, **H5**	2 *Ld*
10	SJ-LP	−31.476	−67.351	**H8**, *H38* (cpH4) ; **H3**, *H32*	3 *Lc,* (1 *Lc*); 1 *Ld*
11	SJ-CA	−31.878	−68.525	**H11**, **H12**, **H13**, H19, *H39* (cpH4) ; **H3**(cpH2)	3 *Lc,* (1 *Lc*); 2 *Ld* (1*Ld*)
12	SL-JU	−32.532	−65.329	**H3**, H54 ( cpH 2)	1 *Ld* (1 *Ld*)
13	ME-US	−32.568	−69.340	**H8**, **H9**;**H3**, *H32* ( cpH 2) ; H20, *H40, H41* (cpH6)	1 *Lc*; 3 *Ld,* (1 *Ld*); 2 *Ln,* (1 *Ln*)
14	SL-SQ	−32.899	−66.740	**H8**, **H9**(cpH4) ; **H3**( cpH 2)	2 *Lc,* (3 *Lc*); 2 *Ld,* (4 *Ld*),
15	ME-LC	−33.079	−69.065	**H3**, **H4**, **H5**( cpH 2)	3 *Ld,* (1 *Ld*)
16	ME-GA	−33.362	−68.266	**H7**(cpH4) ; **H3**, *H35* ( cpH 2, cpH3)	2 *Lc,* (2*Lc*); 3 *Ld,* (2 *Ld*)
17	ME-RG	−36.313	−69.666	**H3**, H16	2 *Ld*
18	LP-ED	−36.708	−65.289	**H5**	2 *Lc*
19	LP-LR	−37.557	−66.233	**H3**, H1 6	2 *Lc*
20	RN-CTN	−37.900	−67.862	**H8**, **H9**;**H3**, *H37*	1 *Lc*, 3*Ld*
21	RN-CTS	−38.057	−67.928	( cpH 2)	(1 *Ld*)
22	LP-LC	−38.136	−65.591	**H3, H5, H7 ,** *H34*, ( cpH 2) ; (cpH4)	2 *Ld*, (5 *Ld);* (4 *Ln*)
23	NE-CH	−39.243	−68.974	**H7**, **H10**(cpH4) ; **H3**, **H5**(cpH2, cpH3) ; (cpH4)	2 *Lc,* (1 *Lc*); 2 *Ld,* (3*Ld*); (2 *Ln*)
24	RN-VA	−40.416	−66.139	**H8**(cpH4) ; **H3**, **H5**, *H32*	3 *Lc,* (2 *Lc*); 3 *Ld*
25	RN-SC	−40.583	−67.800	(cpH4)	(2 *Ln*)
26	RN-SA	−40.683	−65.133	(cpH4)	(3 *Ln*)
27	RN-LG	−40.883	−65.133	**H3, H6** (cpH2) ; H23, *H48* (cpH5)	2 *Ld* (2 *Ld*); 1 *Ln* (3 *Ln*)
28	RN-MA	−40.961	−68.440	**H14**, H24, *H49* (cpH6) ; H20, H21, H22, *H45, H46, H47* (cpH4, cpH6)	3 *La* (3 *La*); 3 *Ln* (5 *Ln*)
29	RN-RN3	−41.016	−65.383	**H14** (cpH6) ; H **8**, H **9**, H38 ; *H35*; H20, H21, *H42, H43* (cpH4) ; H14 **, H15,** H25, *H50, H51, H52, H53*	3 *La* (1 *La*); 4 *Lc;* 2 *Ld;* 2 *Ln,* (2 *Ln*), 6 HS
30	RN-SP	−41.184	−65.968	H16 , *H36 IV*; H26, *H44*	1 *Ld*; 1 *Ln*
31	CH-MA	−43.515	−68.180	(cpH2) ; (cpH4)	(4 *Ld*); (5 *Ln*)

Color-code: *L. ameghinoi* (*La*): in pink; *L. cuneifolia* (*Lc*): in green; *L. divaricata* (*Ld*): in red; *L. nitida* (*Ln*): in orange, *L. tridentata* (*Lt*): in yellow, Hybrid Swarm (HS):in cyan.

### DNA extraction and sequencing

To test the ability of barcode markers to solve the phylogeny of the genus *Larrea*, we extract DNA and amplified two molecular markers, nuclear ITS2, hereafter ITS, and the plastid gene rbcL, at the Molecular Laboratory of INIBIOMA in Argentina according to the protocol of Canadian Center for DNA Barcode (Ivanova et al. 2008). We used 40 mg of dehydrated leaf tissue of plants collected in the field to extract total genomic DNA using the alkyl-trimethyl-ammonium bromide method modified for samples high in polysaccharides (Doyle and Doyle 1990). The ITS region was amplified with primers ITS_S2F (Chen et al. 2010) and ITS4 (White et al. 1990), while the rbcL gene, was amplified with the primers rbcLa-F (Levin et al. 2003) and rbcLa-R (Kress and Erickson 2007) (Supplementary Table S2). These nuclear and chloroplast markers have been extensively used in phylogenetic studies of the Zygophyllaceae Family (Bellstedt et al. 2012; Wu et al. 2015, 2018; Godoy-Bürki et al. 2018; Böhnert et al. 2020; Selvaraj and Ramalingam 2021), and in previous attempts to solve *Larrea* species relationships (Lia et al. 2001; Laport et al. 2012). Our samples were either sequenced at the Canadian Centre for DNA Barcoding, as part of the Barcoding Plant Species from the Monte Desert, BOLD project (https://portal.boldsystems.org/), or at Macrogen, Korea. To complement our analyses, we downloaded sequences of sister genera *Bulnesia* (three species), *Fagonia* (three species), and *Guaiacum* (two species), along with sequences of *Tribulus* (one species) and Zygophyllum (five species) that were used as external groups (Supplementary Table S3).

### Haplotype alignment and network analyses

When visualizing chromatograms for the nuclear region ITS, we observed two different types of sequences, ‘pure haplotypes’ with no ambiguities at any base pair and with a particular combination of base-pairs for a given species; and sequences that showed double peaks (Supplementary Fig. S1) for Schematic chromatograms showing alternative types of sequences, with two putative genetic bases per variable site, hereafter named ‘putative haplotypes’ (PH). As previously observed by Hunter (1995), we obtained double-stranded DNA of ITS for many individuals, identifying more than one sequence for the same individual. Such sequences were considered two PHs for the same individual (Supplementary Data). One sequence showed a base variant that can be assigned to a nominal species (PH1), while the other haplotype contains the putative alternative base (PH2). Meanwhile, rbcL sequences showed regular chromatograms. We aligned all sequences manually using Mega v.7.0.14 (Kumar et al. 2016).

We constructed Median-joining haplotype networks (Bandelt et al. 1999) for each marker independently implemented in NETWORK 5.0.0.3. (https://www.fluxus-engineering.com/). To disentangle the complex relationships among ITS haplotypes, we built up three networks: one including pure haplotypes, a second network of pure and PH1 haplotypes, and a third network including pure, PH1, and PH2 haplotypes. We also mapped populations’ haplotypes of both ITS and rbcL sequences in QGis 3.14 (QGIS Geographic Information System 2015), using field geo-references (Table 1).

### Phylogenetic analyses and preliminary molecular dating

To reconstruct relationships among ITS haplotypes, we ran three independent Bayesian phylogenetic trees: one including pure haplotypes, a second tree of pure and PH1, and a third tree including pure, PH1, and PH2. Evolution models that best fit these trees were GTR + G + I (Rodriguez et al. 1990), setting tree prior clock with a strict model, and speciation as Yule Process. The analysis consisted of 10 000 000 generations and trees were saved every 1000 generations in each run. To estimate divergence ages among haplotypes for each marker, we ran the above-mentioned ITS trees, and a rbcL haplotype tree. We used BEAST v.1.7.2 (Drummond et al. 2012), with a model of sequence evolution generated by jModelTest v.2.1.5 (Darriba et al. 2012) that implemented the substitution evolutionary model with AIC. We verified the convergence of the estimated parameters using Tracer v.1.4 (Rambaut and Drummond 2008). Standard deviations of former calibrations yielded similar 95% confidence intervals. We used TreeAnnotator (Helfrich et al. 2018), summarizing mean heights, excluding a burn-in of 10%, and a posterior probability limit of 0.95 to obtain high posterior density (HPD) intervals only for nodes of interest with sufficient support.

We calculated divergence ages, applying a Bayesian lognormal relaxed clock (uncorrelated), including a birth-death process *prior* to the speciation process (following Böhnert et al. 2020 and; Wu et al. 2015). We set 100 million generations runs (sampling every 10 000 generations and 10% discarded as burn-in), using subsequent trees to estimate Bayesian posterior probabilities in each consensus tree. We calibrated trees with secondary calibration points based on Wu et al. (2015), constraining the root age and estimating node ages following normal prior distributions with a mean of 67.7 Myr (sd = 10) for clade Larreoideae + Zygophylloideae, a mean of 25 Myr (sd = 7) for Larreoideae clade, and a mean of 18 Myr (sd = 5) for clade *Larrea* (Supplementary Table S4).

## Results

### Nuclear ITS marker: haplotype variation and network analyses

The ITS matrix included 111 sampled individuals and a total length of 316 bp, with 61 polymorphic sites (55%), 25 of these sites exhibited double peaks, which were interpreted as putative sequences for the same individual. This resulted in 178 ITS sequences, totalling 56 distinct haplotypes, including 15 pure haplotypes, 11 PH1, and 30 PH2. The 61 polymorphic sites in this marker allowed us to genetically distinguish pure haplotypes for each taxon using 18 informative sites, detect intraspecific variation with another set of 21 sites, and differentiate PH through other 22 sites with ambiguities (Supplementary Data). The 15 pure haplotypes obtained were: two for *Lt* (H1 and H2); four for *Ld* (H3–H6); seven for *Lc* (H7–H13); one haplotype for *La* (H14), and one for HS (H15) (Table 1; Fig. 1A; Supplementary Data). *Ln* individuals showed sequences with double peaks at informative sites, however, the putative H20 was only present in *Ln* individuals so it was included when building the pure haplotypes network (Fig. 1B). This network, clearly differentiated the six studied taxa, except for H3 which is an *Ld* haplotype, also present in one individual morphologically identified as *Lc* in a sympatric population of these two species (population SA-CA). This network also revealed that bifoliated species were more closely related among them than with multifoliated ones. This was reflected in the closer association between *Lt* haplotypes to the most common haplotype H3 of *Ld*, as well as the higher number of changes separating the bifoliated *Lc* and the multifoliated taxa (*La*, *Ln*, and HS) (Fig. 1B).

**Figure 2.**
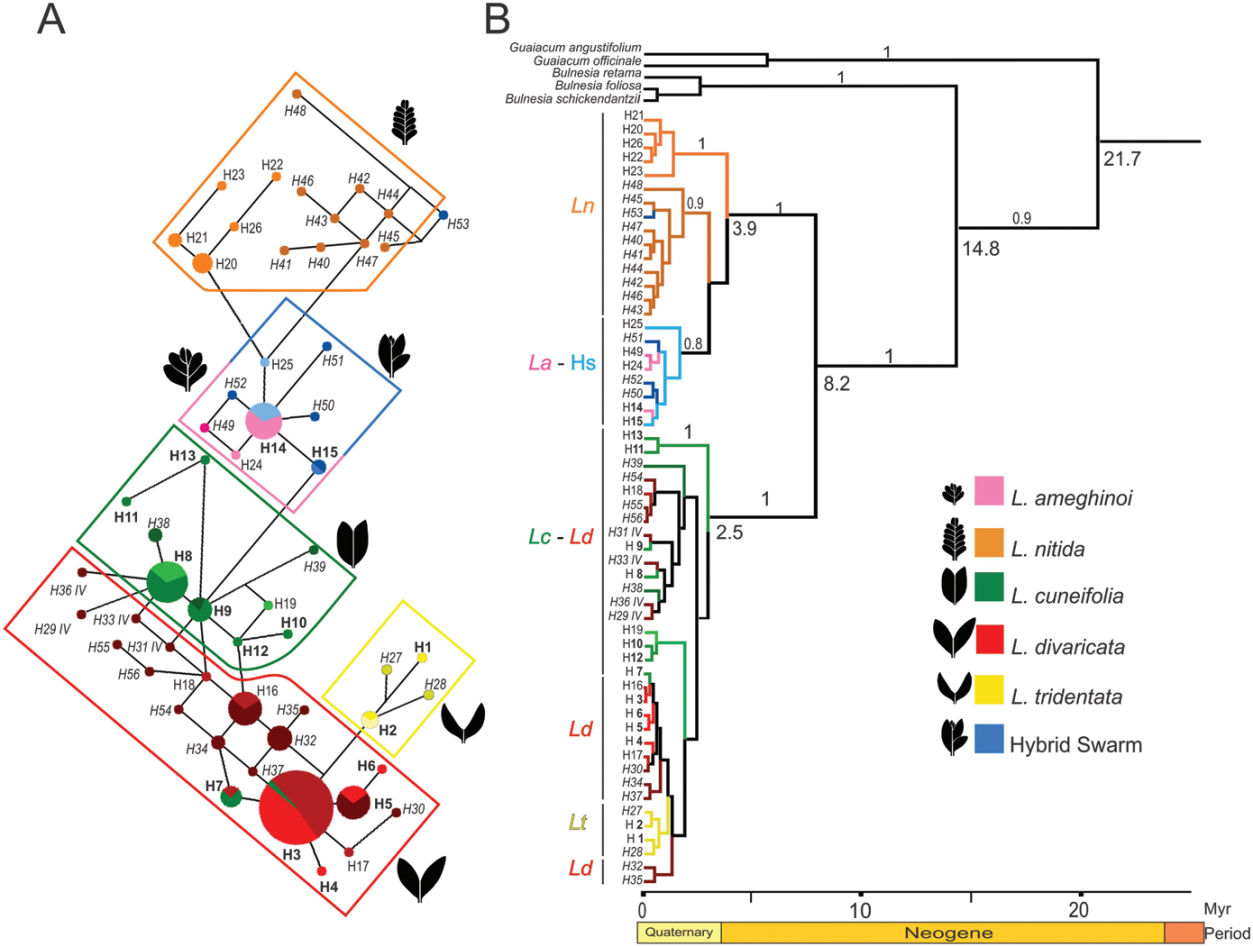
(A) ITS pure and PH network. Circle size is proportional to haplotype frequencies, and between-haplotype branch length is proportional to the number of mutational changes. Pure haplotypes are shown in bold. Putative haplotypes (PH1) are represented in lighter colours, and PH2 are represented in darker colours and italics. Hybrid, i.e. interespecific variants are indicated *as*  *IV*. (B) Calibrated phylogenetic relationships among *Larrea* species, with Bayesian posterior probabilities below each branch. On intersections, estimated ages were calculated using BEAST. Colours correspond to morpho-species and next to it, species-specific leaf morphology. Colour codes: *La* in pink range, *Lc* in green range, *Ld* in red range, *Ln* in orange range, *Lt* in yellow range, and HS in cyan range.

Subsequently, when we added the 11 PH closer to each nominal species (i.e. PH1) to the network analysis, we observed eight PH1 (H17, H19, and H21–26) derived from their pure parental haplotypes of *Ld*, *Lc*, *Ln*, *La*, and HS, considered within-species genetic variation (Supplementary Fig. S2). Two PH1 (H16 and H18) observed in this network showed signs of hybridization between species. Both were observed in *Ld* individuals, and each was connected with one pure *Lc* haplotype, H12 and H9, respectively. Surprisingly, we also detected two shared haplotypes between allopatric pairs of taxa, H7, a pure *Lc* haplotype, was detected as a PH in one individual morphologically identified in the field as *Ld*. Likewise, H14, a pure *La* haplotype, was found as a PH in four sympatric individuals identified in the field as HS (Supplementary Data; Supplementary Fig. S2). The network became a grid when we included the 30 haplotypes recorded as putative sequences (PH2) which were H27 to H56. Four of these haplotypes were interspecific variants (*IV*), that were closer in the network with common pure haplotypes of other species rather than the nominal species observed in the field, i.e. H29, H31, H33, and H36 *Ld* haplotypes were closer to H8 and H9 *Lc* pure haplotypes than to H3 *Ld* pure haplotype. Also, we observed 26 derived PH2 haplotypes that are peripheric in the network and considered intraspecific variation (Fig. 2A).

### Haplotype phylogenetic analyses

We performed haplotype phylogenetic analyses following the same stepwise approach: first, including only pure haplotypes; second, adding the putative haplotypes PH1; and finally, including PH2 haplotypes. We obtained an ITS phylogeny with two main clades when we considered only pure haplotypes. One included multifoliated species haplotypes: *La*, *Ln,* and HS (the molecular variants of the morphological hybrids). The other clade included bifoliated species haplotypes: *Lc*, *Ld,* and *Lt* where *Lt* formed a monophyletic clade, and *Lc* and *Ld* were paraphyletic (Fig. 1C). We detected several nuclear lineages for *Lc* (Fig. 1C): one, including H11 and H13, that diverged earlier than the others, and a second lineage, including H8 and H9. The third lineage (H10 and H12) diverged later in the nuclear phylogeny closely related to the *Ld* and *Lt* haplotypes. Finally, the lineage including *Lc* haplotype H7 clustered with *Ld*, since these two species shared species-specific base pairs in this haplotype (Supplementary Data). When we added the first 10 alternative variants, H16 and H17 *Ld* PH1 haplotypes, nested with *Ld* pure haplotypes, however H18 *Ld* pure haplotypes nested with H8 and H9 *Lc*. The only PH1 of *Lc*, H19, nested with its pure haplotypes H10–H12. *Ln* showed a monophyletic clade including haplotypes H20–22 and H26. *La* and HS haplotypes formed one clade including H14–15 and H24–25 (Supplementary Fig. S2B). When also including PH2, seven haplotypes were nested independently of their nominal species (Fig. 2B), and of them (H29, H31, H33, and H36) were *Ld* haplotypes nesting in the H8–H9 *Lc* clade, and were considered interspecific variation (*IV*). Other three *Ld* PH2 haplotypes, H54–56, nested in one clade sister to a *Lc* clade. Then, *Ld* PH2 haplotypes (H30, H32, H34–35, and H37) were nested with *Ld* pure haplotypes. *Lc* showed two PH2 haplotypes (H38–39), that nested with *Lc* pure haplotypes. Meanwhile, *Ln* haplotypes were paraphyletic, since all *Ln* PH1 form one clade, and PH2 of *Ln* and H53 of HS, were nested in other clade, sister to a *La*–Hs clade, which included pure, PH1, and PH2 variants (Fig. 2B).

### Variation in the chloroplast rbcL maker

**Figure 3:**
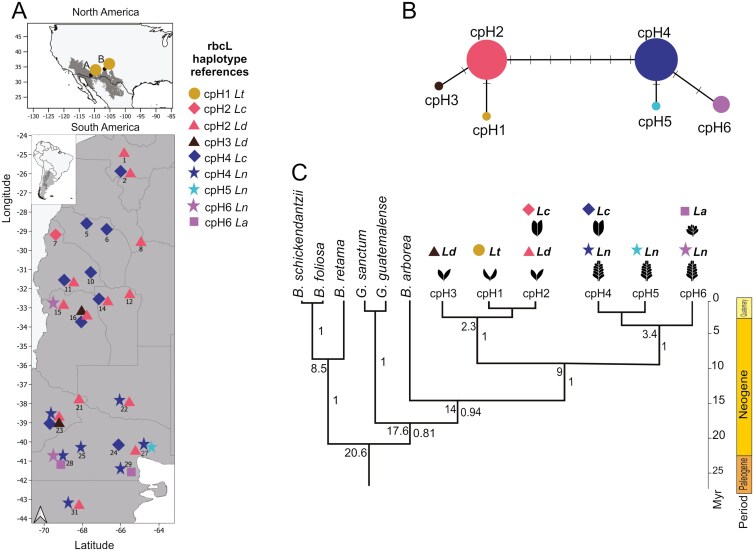
(A) Sampled locations of *Larrea* species rbcL gene haplotype in North and South America; population reference as in Table 1. Forms (*Lt* circle, *Lc* diamond, *Ld* triangle, *Ln* start, *La* square) and colours depict haplotypes at each location. (B) rbcL gene haplotypes network: circle size proportional haplotype frequencies and between-haplotype branch length is proportional to the number of changes between haplotypes, and (C) rbcL calibrated haplotype network and phylogeny: phylogeny of haplotypes, numbers on the right of the branches represent Bayesian posterior probabilities. Estimated ages (Myr), calculated using BEAST, are shown on intersections. Colour codes correspond to: cpH1 in ochre, cpH2 in pink, cpH3 in black, cpH4 in blue, cpH5 in cyan, and cpH6 in violet.

The chloroplast rbcL alignment included 84 sequences corresponding to *La* (*N* = 4), *Lc* (*N* = 35), *Ld* (*N* = 18), *Ln* (*N* = 23), and *Lt* (*N* = 4). For the rbcL gene, we obtained an alignment of 568 bp that contained 12 informative sites (2%), resulting in a total of six haplotypes labelled as cpH_1 to cpH_6 (Table 1; Fig. 3A). Despite our efforts, we were unsuccessful in obtaining PCR amplification products for the rbcL plastid region of HS individuals. The six rbcL haplotypes were shared by three species, mainly allopatric pairs, *Ln*–*Lc*, *Ld*–*Lc*, and *La*–*Ln* (Fig. 3B). The rbcL haplotypes formed two haplogroups, one containing bifoliated species consisting of cpH_1, recorded for *Lt*, cpH_2 commonly found in many localities of *Ld* and one non-sympatric *Lc* individual, and cpH_3 that was observed only in *Ld* individuals. The other haplogroup comprises multifoliated species, a widespread cpH_4 observed in *Lc* and *Ln*, although sympatric in only one population (Neuquen—El Chocón), cpH_5 species-specific of *Ln* samples, and cpH_6 presented in non-sympatric *Ln* and *La* individuals (Fig. 3C). For the rbcL gene *Ln* was the most polymorphic and monophyletic species. In addition, neither *Lc* nor *La* showed species-specific chloroplast haplotypes. Surprisingly, the chloroplast yielded retention of widespread ancient polymorphisms, which were conserved in populations of distinct species in sympatry and parapatry.

### Molecular dating

Since the nuclear and plastid haplotype phylogenies were slightly incongruent, we dated them independently. Nonetheless, we obtained a similar point estimate for the crown age of *Larroideas* of 21.7 Myr (95% HPD: 10.1–24.5); and 20.6 Myr (95% HPD: 7.4–20.6) for ITS and rbcL, respectively (Figs. 2B and 3C). For the nuclear region, we observed two distinguished clades, the multifoliated clade including *La, Ln*, and the HS, and a second bifoliated clade containing *Ld, Lc,* and a monophyletic subclade of *Lt* haplotypes. The divergence between the two clades of the nuclear region phylogeny occurred approximately 8 Myr ago (95% HPD: 5.4–13.5) (Fig. 2B). Similarly, for the chloroplast region, the divergence between the clade including the multifoliated *La*, *Ln*, and bifoliated *Lc* and the clade with only bifoliated species *Ld*, *Lc*, and *Lt*, occurred approximately 9 Ma ago (95% HPD: 0–18) (Fig. 3C). The diversification of the *Larrea* clades occurred first for the clade containing the multifoliate *La* and *Ln* (3.9 and 3.4 Ma ago for ITS and rbcL, respectively), and after for the other species (2.5 and 2.3 Ma ago for ITS and rbcL, respectively; Figs. 2B and 3C). Thus, despite the phylogenetic discordance regarding *Lc* for different markers, divergence ages between main clades were similar for both markers, also showing high posterior support (Table 2) (Supplementary Figs. S3 and S4, respectively for ITS and rbcL phylogenies and their calibrating points (Supplementary Tables S5 and S6).

**Table 2.** Estimated ages of divergence, in million years ago, for the ITS nuclear region and rbcL gen, estimated using BEAST.

Taxon	ITS	rbcL
Root (Tribulus)	60 (94–30)	60 (94–30)
Zygophylloide	44.2 (67–41)	44 (67–41)
Larroidea	21.7 (29–12)	20.6 (29–12)
*Larrea*	8.2 (13.5–5.4)	9 (18–0)
Clade *Lc, Ld, Lt*	2.5 (5.1–1.9)	–
Clade cpH 1, 2, 3	–	2.5 (6–0)
Clade *La, Ln*, HS	3.9 (6.6–2.5)	–
Clade cpH 4, 5, 6	–	3.4 (8–0)

## Discussion

The exhaustive analysis of DNA sequences of two independent markers of several individuals of different *Larrea* species from multiple localities across their ranges showed that some topological discordance exists between nuclear and chloroplast markers. The nuclear ITS data support the monophyly of the bifoliated and multifoliated sections within the genus. Also, only geographically restricted species, i.e. *Lt* from North America and *La* from South America resulted as monophyletic whereas more widespread species, *Ln*, *Lc,* and *Ld* were paraphyletic. In contrast, the chloroplast marker, rbcL, unequivocally yielded a widespread gene pool consisting of divergent variants, which were conserved through time in populations of distinct species. The most polymorphic species for the rbcL was *Ln,* despite its relatively more restricted range. Surprisingly, the most widespread variant of the bifoliated wide-ranging *Lc* yielded a close relation to the mutlifoliated *Ln*. We identified two key evolutionary processes that might explain these relationships, ancient and still ongoing hybridization in the nuclear marker, and retention of ancient polymorphisms yielded by the chloroplast. These processes are facilitated by a particular set of life history traits that characterize *Larrea* species, i.e. pollinator territorial behaviour and local seed dispersal with chloroplast paternal inheritance piles up with high levels of self-pollination (Tadey et al. 2009; Tadey 2015). Although such traits may foster between-species coherence that allegedly drove adaptation and radiation within this genus, synchronic flowering phenology under water-stressed limited conditions may promote interspecific gene flow (Hunziker et al. 1977; Yang et al. 1977, 2000; Tadey 2020). Therefore, when analysing the two barcode marker sequences and mapping the haplotypes distribution we contributed to disentangle the phylogenetic relationships and evolutionary history of the widespread amphitropical genus *Larrea*.

### Hybridization and retention of ancestral polymorphisms as sources of evolutionary novelties

Hybridization is common among certain plant lineages and is known to produce evolutionary novelties, by combining parental genomes in adaptive ways, operating as modulators, and perhaps a catalyst of speciation in the Neotropical flora (Rieseberg et al. 2007; Cuevas et al. 2022; Schley et al. 2022). Interspecific hybridization is a process in which closely related species interbreed, producing offspring with admixed genomes (Runemark et al. 2019). Our results confirmed early morphological and cytogenetic studies that proposed hybridization among *Larrea* species (Hunziker et al. 1969, 1978). As previously observed by Hunter (1995), we obtained double-stranded DNA of ITS for many individuals, identifying more than one sequence for the same individual, which had been reported as sources of polymorphism and reticulation in other angiosperms such as *Cedrela* sp. (Garcia et al. 2011; Quiroga et al. 2016), members of the genus *Passiflora*, and other herbaceous species (Aguilar and Feliner 2003; Lorenz-Lemke et al. 2005; Muschner et al. 2006; Hürkan and Taskin 2021). In concordance with these authors, we reported the complete overlap of chromatogram peaks and the decrease in peak intensity to approximately half of the adjacent peaks (Supplementary Fig. S1). As these authors, we interpreted such chromatogram patterns as a reflection of intraspecific variation (i.e. PH) and hybridization (i.e. interspecific variation). Analyzing many individuals from different populations and different species and considering pure haplotypes and double peaks in chromatograms as PH, we were able to differentiate intraspecific variation from hybridization, assigning PH to one or the other species involved in the hybridization process. Double peaks in *Lc* and *Lt* were not a surprise since both species are polyploids. Interestingly, we detected interespecific genetic variants *IV* in individuals morphologically assigned unequivocally to one species. These genetic variants occurred at relatively low frequencies (i.e. in one or two individuals per population), but we observed them in most populations, reflecting ancient local hybridization that is not reflected in the morphology. Such cryptic hybridization suggests that morphological identification in the field underestimates the extent of genetic variation in this genus. Cryptic hybridization has been observed also in *Protea* sp. and pinyon pines (Mitchell and Holsinger 2018; Buck et al. 2020). Surprisingly, the hybrid swarm HS, which consist of morphological hybrids, showed specific haplotypic variants, but we only observed them in one population, where all four Southern South American *Larrea* species co-occur, probably due to *La* ability to hybridize, as suggested by Hunziker et al. (1977).

Shared ancestral cpDNA haplotypes have been reported in angiosperms (Choi et al. 2021; Li et al. 2022; Stull et al. 2023). We observed evidence that *Lc* chloroplasts are paraphyletic, occurring in two different chloroplast lineages, suggesting retention of ancestral polymorphisms. *Larrea* is successful in circumventing different steps that lead to the exclusion or degeneration of paternal plastids or the degradation of paternal plastid DNA during the development of the male gamete and its passage to the egg cell, and further during or after fertilization (Yang et al. 2000). Paternal inheritance of cpDNA in *Larrea* species might favour chloroplast conservation and, therefore, retention of ancestral chloroplasts. Polymorphisms are often studied in a single species, while closely related species with similar polymorphisms are usually overlooked. Here we show how the study of related species and multiple populations allows a broad evolutionary interpretation of the possible origins of the observed plastid polymorphisms. A macroevolutionary perspective on polymorphisms will bring insights into the varied roles of polymorphisms in evolution and deepen our understanding of the conditions under which polymorphisms persist through speciation (Soltis et al. 2015; Li et al. 2022). Analyzing nuclear DNA and chloroplast markers we disentangle *Larrea* species phylogeny, although they lead to the classic cyto-nuclear discordances in haplotype phylogenies. Such a pattern is expected when the processes occurring are independent and under different evolutionary pressures (Acosta and Premoli 2010; Premoli et al. 2011).

### Haplotypic phylogenetic inference and divergence times

Zygophyllaceae *sensu lato* is a widespread family of trees, shrubs, sub-shrubs, and herbs, mostly restricted to arid and semi-arid areas in the tropics and subtropics (Hunter 1995; Roig et al. 2009). In general, we obtained a phylogenetic reconstruction for this family (Supplementary Figs. S3 and S4), similar to the previously published cpDNA Bayesian inference models of Böhnert et al. (2020), and the one obtained using ITS and chloroplast regions analysed via Bayesian inference and Maximum likelihood by Wu et al. (2015). In this study, we revisited the phylogeny of the genus *Larrea* to solve a long-lasting riddle, including many populations and individuals per species. Although we obtained relatively incongruent haplotype trees for two independent markers, the divergence time between their two main clades was consistent (8–9 Myr ago). As Roig et al. (2009) suggested, *Larrea* is a recently evolved genus, with diversification time within the late Miocene (11.6–9.0 Myr). During this period, global open lands continued to expand and forests dwindled in extent, as the climate turned drier. Well-studied continental exposures occur in the North American Great Plains and Argentina (Ramos 1999; Graham 2009) and congruent divergence times between both markers suggest that retention of ancestral chloroplasts and nuclear hybridization processes might have occurred synchronously. One caveat to consider is the uncertainty in the estimated divergence times, due to large variation of the fossil calibration record, limited cpDNA variation, and some inherent problems of nuclear data (i.e. uncertain mutation model and homoplasy; Selkoe and Toonen 2006).

Focusing on the genus *Larrea*, several phylogenetic studies have been published since the 70s (Hunziker et al. 1972; Hunziker 1975; Wells and Hunziker 1976; Poggio et al. 1989; Hunter 1995; Lia et al. 2001; Laport et al. 2012). We built up on these studies to disentangle the long-lasting riddle of phylogenetic relationships in this amphitropical genus. For the ITS phylogeny we obtained two main clades, one including *Ln*, *La,* and the HS, as sister to a clade with *Lc*, *Ld,* and *Lt* haplotypes. This classification agrees with the one obtained by Palacios and Hunziker (1972), Hunziker et al. (1977) based on leaflets morphology and cytogenetic studies, and the more recent nuclear phylogeny reported by Laport et al. (2012), although the latter focussed on *Lt* and omitted *La*. Our nuclear results were consistent with the hypothesis that the multifoliated section *Larrea* diverged earlier than the bifoliated section, *Bifolium*, and that *Lt* forms a monophyletic group, sister to *Ld*. However, Hunziker et al. 1977, based on cytogenetic evidence suggested that *Lc* and *Ld* are closely related to the extent that *Ld* (or an extinct species closely related to it) has been one of the progenitors of the tetraploid *Lc*. Meanwhile, in our results, *Lc* occurred in a more internal position of the phylogeny, while *Ld* and *Lt* were more external, suggesting *Lc* as an early-diverging lineage within the bifoliated clade.

The incongruence between markers in our results arises because the bifoliated *Lc* clustered with the other bifoliated species in the nuclear phylogeny but with the multifoliated species in the chloroplast phylogeny, as observed by Lia et al. (2001) and by Laport et al. (2012). These authors concatenated nuclear and chloroplast markers and justified such incongruence via intersectional hybridization (Supplementary Table S1). Lia et al. (2001) interpreted this as the result of the reticulate allopolyploid origin of *Lc*. However, such incongruence might have arisen from horizontal transfer, lineage sorting, gene duplication, and extinction (Doyle 1997; Maddison 1997; Slowinski and Page 1999). We found evidence of hybridization in the studied nuclear region (between *Ld*–*Lc*), along with retention of ancestral polymorphism in the shared chloroplasts (between *Lc*–*Ln, Ln*–*La,* and *Lc*–*Ld*). Our chloroplast haplotype tree showed two sister clades that discriminated bifoliated of *Ld*, *Lt*, and *Lc* haplotypes from the other clades, including multifoliated *Larrea* species and *Lc* haplotypes. Thus, our results suggested that *Lc* might have retained the chloroplast of the ancestor of both sections rather than giving an idea of the origin of this tetraploid. Life history traits of the genus *Larrea*, paternally inherited chloroplasts, restricted pollen and seed dispersal, the ability to form inter-fertile hybrids between species of different ploidy levels occurring in sympatry, and their widespread range across disjunct arid and semiarid environments, constitute an interesting and complex combination of characteristics to study plant abilities to survive harsh environments.

Vidal-Russell et al. (2022) estimated the relationship between the relative DNA content of *Larrea* species and environmental variables and found both intra- and interspecific variation in genome size. As previously observed by Laport et al. (2012), *Larrea tridentata* showed a gradient of increasing auto polyploidy (2x, 4x, and 6x) suggesting that environmental pressures may have facilitated repeated whole genome duplication events in North America. Meanwhile, three South American species were diploids (*Ld*, *Ln*, and *La*), and *Lc* was an allopolyploid. This was considered an indication that in South America, reticulate evolution, as allopolyploidization, and speciation might have been climate-dependent since the Oligocene.

### Barcode use

This study used barcode data to address a phylogenetic problem. The widely available barcode data can also provide insights into hybridization, interspecies chloroplast capture, or the retention of ancestral chloroplasts, as well as recent speciation events driven by polyploidization and shifts in breeding systems (de Vere et al. 2015; Batley 2016; Kartzinel et al. 2025). Phylogenetic reconstructions and phylogeographic tools, particularly those based on genetic barcodes, can provide valuable insights into the processes that have influenced the genetic makeup of lineages. However, these tools also have limitations that must be considered in the interpretation of evolutionary patterns. When multiple co-distributed taxa and populations are included, these methods can deepen our understanding of the causal relationships between species distributions and the mechanisms driving speciation (Avise et al. 1987). Future studies incorporating genomic markers, such as whole-genome sequencing or transcriptomics, could further unravel complex phylogeographic questions by providing more detailed insights into genetic variation and evolutionary processes.

## Concluding remarks

Barcode sequences aid to disentangle the phylogeny of the amphitropical genus *Larrea*, suggesting the pattern ‘everything, everywhere, all at once’ as a result of hybridization and retention of ancestral chloroplast polymorphisms. Such drivers of diversification might give a clue to other unsolved phylogenies. Shared ancestral chloroplast variants and hybridization are more common than recognized in plants and might be key sources of genetic variation under unfavourable environments. Barcoding plant species can contribute to understanding the strategies that give plant species resilience to face past environmental changes and allow us to predict alternative evolutionary responses of species. We recommend caution when using a combination of barcoding genes in related plant species since they might reflect reticulated processes. These extended phenomena in plants maintained evolutionary lineages with a genetic mosaic and gave them resilience to face the changes that have occurred on the planet over evolutionary time.

## Supplementary Material

plaf024_suppl_Supplementary_Figures

plaf024_suppl_Supplementary_Tables

plaf024_suppl_Supplementary_Data

## Data Availability

Find sampled individuals, species, localities, haplotype frequencies, and GeneBank accession numbers in Supplementary Tables S7 and S8.

## References

[CIT0001] Acosta MC, Premoli AC. Evidence of chloroplast capture in South American *Nothofagus* (subgenus *Nothofagus*, Nothofagaceae). Mol Phylogenet Evol 2010;54:235–42. 10.1016/j.ympev.2009.08.008 19683588

[CIT0002] Aguilar JF, Feliner GN. Additive polymorphisms and reticulation in an ITS phylogeny of thrifts (*Armeria*, Plumbaginaceae). Mol Phylogenet Evol 2003;28:430–47. 10.1016/S1055-7903(02)00301-9 12927129

[CIT0003] Amarilla LD, Chiapella JO, Sosa V et al A tale of North and South America: time and mode of dispersal of the amphitropical genus *Munroa* (Poaceae, Chloridoideae). Bot J Linn Soc 2015;179:110–25. 10.1111/boj.12304

[CIT0004] Avise JC, Arnold J, Ball RM et al Intraspecific phylogeography: the mitochondrial DNA bridge between population genetics and systematics. Annu Rev Ecol Syst 1987;18:489–522.

[CIT0005] Bandelt H-J, Forster P, Röhl A. Median-joining networks for inferring intraspecific phylogenies. Mol Biol Evol 1999;16:37–48. 10.1093/oxfordjournals.molbev.a026036 10331250

[CIT0006] Batley J. Plant Genotyping. Methods and Protocols. NY: Springer. p. 315, 2016. 10.1007/978-1-4939-1966-6

[CIT0007] Bellstedt DU, Galley C, Pirie MD et al The migration of the palaeotropical arid flora: Zygophylloideae as an example. Syst Bot 2012;37:951–9. 10.1600/036364412x656608

[CIT0008] Böhnert T, Weigend M, Merklinger F et al Historical assembly of Zygophyllaceae in the Atacama Desert. Front. Biogeogr 2020;12:e45197. 10.21425/F5FBG45197

[CIT0009] Buck R, Hyasat S, Hossfeld A et al Patterns of hybridization and cryptic introgression among one-and four-needled pinyon pines. Ann Bot (Lond) 2020;126:401–11. 10.1093/aob/mcaa045 PMC7424738 32222765

[CIT0010] Burnham RJ, Graham A. The history of neotropical vegetation: new developments and status. Ann Mo Bot Gard 1999;86:546–89. 10.2307/2666185

[CIT0012] Chen S, Yao H, Han J et al Validation of the ITS2 region as a rovel DNA barcode for identifying medicinal plant species. PLoS One 2010;5:e8613. 10.1371/journal.pone.0008613 20062805 PMC2799520

[CIT0011] Choi JY, Dai X, Alam O et al Ancestral polymorphisms shape the adaptive radiation of *Metrosideros* across the Hawaiian Islands. Proc Natl Acad Sci USA 2021;118:e2023801118. 10.1073/pnas.2023801118 34497122 PMC8449318

[CIT0013] Cuevas A, Eroukhmanoff F, Ravinet M et al Predictors of genomic differentiation within a hybrid taxon. PLoS Genet 2022;18:e1010027. 10.1371/journal.pgen.1010027 35148321 PMC8870489

[CIT0014] Darriba D, Taboada GL, Doallo R et al jModelTest 2: more models, new heuristics and parallel computing. Nat Methods 2012;9:772. 10.1038/nmeth.2109 PMC4594756 22847109

[CIT0015] de Vere N, Rich TC, Trinder SA, et al 2015. DNA barcoding for plants. In: Batley J. (ed.), Plant Genotyping. Methods in Molecular Biology, vol 1245. NY: Humana Press. 10.1007/978-1-4939-1966-6_8 25373752

[CIT0017] Doyle JJ. Trees within trees: genes and species, molecules and morphology. Syst Biol 1997;46:537–53. 10.1093/sysbio/46.3.537 11975332

[CIT0016] Doyle JJ, Doyle JL. Isolation of plant DNA from fresh tissue. Focus 1990;12:13–5.

[CIT0018] Drummond AJ, Suchard MA, Xie D et al; Rambaut. Bayesian phylogenetics with BEAUti and the BEAST 1.7. Mol Biol Evol 2012;29:1969–73. 10.1093/molbev/mss075 22367748 PMC3408070

[CIT0019] Emerson BC. Delimiting species—prospects and challenges for DNA barcoding. Mol Ecol 2025;34:e17677. 10.1111/mec.17677 39912533 PMC11842946

[CIT0020] Fazekas AJ, Kuzmina ML, Newmaster SG, et al 2012. DNA barcoding methods for land plants. In: Kress W., Erickson D. (eds.), DNA Barcodes. Methods in Molecular Biology. vol 858. Totowa: Humana Press. 10.1007/978-1-61779-591-6_11 22684959

[CIT0021] Garcia MG, Silva RS, Carniello MA et al Molecular evidence of cryptic speciation, historical range expansion, and recent intraspecific hybridization in the Neotropical seasonal forest tree *Cedrela fissilis* (Meliaceae). Mol Phylogenet Evol 2011;61:639–49. 10.1016/j.ympev.2011.08.026 21930224

[CIT0022] Godoy-Bürki AC, Acosta JM, Aagesen L. Phylogenetic relationships within the New World subfamily Larreoideae (Zygophyllaceae) confirm polyphyly of the disjunct genus *Bulnesia*. Syst Biodivers 2018;16:453–68. 10.1080/14772000.2018.1451406

[CIT0023] Graham A. The Andes: a geological overview from a biological perspective. Ann Mo Bot Gard 2009;96:371–85. 10.3417/2007146

[CIT0024] Hebert PD, Cywinska A, Ball SL et al Biological identifications through DNA barcodes. Proc Biol Sci 2003;270:313–21. 10.1098/rspb.2002.2218 12614582 PMC1691236

[CIT0025] Helfrich P, Rieb E, Abrami G, et al 2018. TreeAnnotator: versatile visual annotation of hierarchical text relations. In: Proceedings of the Eleventh International Conference on Language Resources and Evaluation (LREC 2018).

[CIT0026] Hollingsworth PM, Graham SW, Little DP. Choosing and using a plant DNA barcode. PLoS One 2011;6:e19254. 10.1371/journal.pone.0019254 21637336 PMC3102656

[CIT0027] Hunter KL. Analysis of the genus *Larrea*: Molecular systematics and polyploidy. UNLV Retrospective Theses & Dissertations. 3001, 1995. 10.25669/29bo-zndi

[CIT0030] Hunziker JH. On the geographical origin of *Larrea divaricata* (Zygophyllaceae). Ann Mo Bot Gard 1975;62:497–500. 10.2307/2395211

[CIT0032] Hunziker JH. Hybridization in Larrea (Zygophyllaceae): a morphological, cytogenetic and chemosystematic study. Bol Acad Nac Cs Córdoba (Argentina) 1978;52:281–314.

[CIT0028] Hunziker JH, Palacios RA, Soriano A. Hibridación natural en especies sudamericanas de *Larrea* (Zygophyllaceae). Kurtziana 1969;5:55–66.

[CIT0029] Hunziker JH, Palacios RA, de Valesi AG, et al 1972. Evolución en el Género *Larrea*. In: Memorias de Simposia, I. Congreso Latinoamericano de Botánica. p. 265–78. México.

[CIT0031] Hunziker JH, Palacios RA, Poggio L, et al 1977. Geographic distribution, morphology, hybridization, cytogenetics, and evolution. In: Creosotebush: Biology and Chemistry of Larrea in New World Deserts. p. 48–91

[CIT0033] Hürkan K, Taskin KM. Internal transcribed spacer (ITS) fails barcoding of the genus Neotinea Rchb. f. (Orchidaceae). J. Agric. Sci 2021;27:69–75. 10.15832/ankutbd.615848

[CIT0034] Ivanova NV, Fazekas AJ, Hebert PDN. Semi-automated, membrane-based protocol for DNA isolation from plants. Plant Mol. Biol. Rep 2008;26:186–98. 10.1007/s11105-008-0029-4

[CIT0035] Kartzinel TR, Hoff HK, Divoll TJ et al Global availability of plant DNA barcodes as genomic resources to support basic and policy‐relevant biodiversity research. Mol Ecol 2025;34:e17712. 10.1111/mec.17712 40018971

[CIT0036] Kress J, Erickson DL. A two-locus global DNA barcode for land plants: the coding rbcL gene complements the non-coding trnH-psbA spacer region. PLoS One 2007;6:1–10. 10.1371/journal.pone.0000508 PMC1876818 17551588

[CIT0037] Kumar S, Stecher G, Tamura K. MEGA7: molecular evolutionary genetics analysis version 7.0 for bigger datasets. Mol Biol Evol 2016;33:1870–4. 10.1093/molbev/msw054 27004904 PMC8210823

[CIT0039] Laport RG, Ramsey J. Morphometric analysis of the North American creosote bush (*Larrea tridentata*, Zygophyllaceae) and the microspatial distribution of its chromosome races. Plant Sys Evol 2015;301:1581–99. 10.1007/s00606-014-1179-5

[CIT0038] Laport RG, Minckley RL, Ramsey J. Phylogeny and cytogeography of the North American creosote bush (*Larrea tridentata*, Zygophyllaceae). Syst Bot 2012;37:153–64. 10.1600/036364412x616738

[CIT0040] Letsiou S, Madesis P, Vasdekis E et al DNA barcoding as a plant identification method. Appl Sci 2024;14:1415. 10.3390/app14041415

[CIT0041] Levin RA, Wagner WL, Hoch PC et al Family-level relationships of *Onagraceae* based on chloroplast rbcL and ndhF data. Am J Bot 2003;90:107–15. 10.3732/ajb.90.1.107 21659085

[CIT0042] Li Y, Wang L, Zhang X et al Extensive sharing of chloroplast haplotypes among East Asian Cerris oaks: the imprints of shared ancestral polymorphism and introgression. Ecol Evol 2022;12:e9142. 10.1002/ece3.9142 35923946 PMC9339761

[CIT0043] Lia VV, Confalonieri VA, Comas CI et al Molecular phylogeny of *Larrea* and its allies (Zygophyllaceae): reticulate evolution and the probable time of creosote bush arrival to North America. Mol Phylogenet Evol 2001;21:309–20. 10.1006/mpev.2001.1025 11697924

[CIT0044] Lorenz-Lemke AP, Muschner VC, Bonatto SL et al Phylogeographic inferences concerning evolution of Brazilian *Passiflora actinia* and *P. elegans* (Passifloraceae) based on ITS (nrDNA) variation. Ann Bot (Lond) 2005;95:799–806. 10.1093/aob/mci079 PMC4246728 15710648

[CIT0045] Maddison WP. Gene trees in species trees. Syst Biol 1997;46:523–36. 10.2307/2413694

[CIT0046] Mitchell N, Holsinger KE. Cryptic natural hybridization between two species of *Protea*. SA J Bot 2018;118:306–14. 10.1016/j.sajb.2017.12.002

[CIT0047] Mogensen HL. Invited special paper: the hows and whys of cytoplasmic inheritance in seed plants. Am J Bot 1996;83:383–404. 10.1002/j.1537-2197.1996.tb12718.x

[CIT0048] Moore AJ, Bartoli A, Tortosa RD et al Phylogeny, biogeography, and chromosome evolution of the amphitropical genus *Grindelia* (Asteraceae) inferred from nuclear ribosomal and chloroplast sequence data. Taxon 2012;61:211–30. 10.1002/tax.611015

[CIT0049] Muschner VC, Lorenz-Lemke AP, Vecchia M et al Differential organellar inheritance in Passiflora’s (Passifloraceae) subgenera. Genetica 2006;128:449–53. 10.1007/s10709-006-7726-4 17028972

[CIT0050] Palacios RA, Hunziker JH. Observaciones sobre la taxonomía del género *Larrea* (Zygophyllaceae). Darwiniana 1972;17:473–6.

[CIT0052] Petit RJ, Excoffier L. Gene flow and species delimitation. Trends Ecol. Evol 2009;24:386–93. 10.1016/j.tree.2009.02.011 19409650

[CIT0051] Petit JR, Duminil J, Fineschi S et al Comparative organization of chloroplast, mitochondrial and nuclear diversity in plant populations. Mol Ecol 2005;14:689–701. 15723661 10.1111/j.1365-294X.2004.02410.x

[CIT0053] Poggio L, Burghardt AD, Hunziker JH. Nuclear DNA variation in diploid and polyploid taxa of *Larrea* (Zygophyllaceae). Heredity 1989;63:321–8. 10.1038/hdy.1989.105

[CIT0089] Premoli AC, Mathiasen P, Cristina Acosta M et al Phylogeographically concordant chloroplast DNA divergence in sympatric Nothofagus s.s. How deep can it be? New Phytologist 2011;193:261–75. 10.1111/j.1469-8137.2011.03861.x 21883239

[CIT0054] QGIS Geographic Information System. Open Source Geospatial Foundation Project. 2015. http://qgis.org

[CIT0055] Quiroga MP, Premoli AC, Grau A et al Local hybridization in subtropical mountain habitats: can *Cedrela* (Meliaceae) maintain species’ identity in sympatry? Drawiniana 2016;4:195–211.

[CIT0056] Quiroga RE, Premoli AC, Fernandez RJ. Climatic niche shift in the amphitropical disjunct grass *Trichloris crinita*. PLoS One 2018;13:e0199811. 10.1371/journal.pone.0199811. 29953506 PMC6023228

[CIT0057] Rambaut A, Drummond AJ. Tracer v1. 4: MCMC Trace Analysis Tool. 2007. Edinburgh: Institute of Evolutionary Biology, University of Edinburgh, 2008.

[CIT0058] Ramos VA. Plate tectonic setting of the Andean Cordillera. Episodes 1999;22:183–90. 10.18814/epiiugs/1999/v22i3/005

[CIT0059] Rieseberg LH, Soltis DE. Phylogenetic consequences of cytoplasmic gene flow in plants. Evol. Trends Plants 1991;5:65–84.

[CIT0060] Rieseberg LH, Kim SC, Randell RA et al Hybridization and the colonization of novel habitats by annual sunflowers. Genetica 2007;129:149–65. 10.1007/s10709-006-9011-y 16955330 PMC2442915

[CIT0061] Rodriguez FJ, Oliver JL, Marín A et al The general stochastic model of nucleotide substitution. J Theor Biol 1990;142:485–501. 10.1016/S0022-5193(05)80104-3 2338834

[CIT0062] Roig FA, Roig-Juñent S, Corbalán V. Biogeography of the Monte desert. J Arid Environ 2009;73:164–72. 10.1016/j.jaridenv.2008.07.016

[CIT0063] Runemark A, Vallejo-Marin M, Meier JI. Eukaryote hybrid genomes. PLoS Genet 2019;15:e1008404. 10.1371/journal.pgen.1008404. 31774811 PMC6880984

[CIT0064] Schley RJ, Twyford AD, Pennington RT. Hybridization: a ‘double-edged sword’ for Neotropical plant diversity. Bot J Linn Soc 2022;199:331–56. 10.1093/botlinnean/boab070

[CIT0065] Selkoe KA, Toonen RJ. Microsatellites for ecologists: a practical guide to using and evaluating microsatellite markers. Ecology Lett 2006;9:615–29. 10.1111/j.1461-0248.2006.00889.x 16643306

[CIT0066] Selvaraj D, Ramalingam S. Identification of morphologically similar species of *Tribulus* (Zygophyllaceae) by employing DNA barcodes and rRNA secondary structures. Ecol. Genet. Genomics 2021;18:100072. 10.1016/j.egg.2020.100072

[CIT0067] Simpson MG, Guilliams CM, Johnson LA. Patterns and processes of American amphitropical disjunctions: new insights. Am J Bot 2017a;104:1597–9. 10.3732/ajb.1700433. https://www.jstor.org/stable/26641682

[CIT0068] Simpson MG, Johnson LA, Villaverde T et al American amphitropical disjuncts: perspectives from vascular plant analyses and prospects for future research. Am J Bot 2017b;104:1600–50. 10.3732/ajb.1700308.

[CIT0090] Slowinski JB, Pages RDM. How should species phylogenies be inferred from sequence data? Syst Biol 1999;48:814–25. 12066300 10.1080/106351599260030

[CIT0069] Soltis DE, Soltis PS, Schemske DW et al Autopolyploidy in angiosperms: have we grossly underestimated the number of species? Taxon 2007;56:13–30. 10.2307/25065732

[CIT0070] Soltis DE, Buggs RJ, Doyle JJ et al What we still don’t know about polyploidy. Taxon 2010;59:1387–403. 10.1002/tax.595006

[CIT0071] Soltis PS, Marchant DB, Van de Peer Y et al Polyploidy and genome evolution in plants. Curr Opin Genet Dev 2015;35:119–25. 10.1016/j.gde.2015.11.003 26656231

[CIT0072] Stull GW, Pham KK, Soltis PS et al Deep reticulation: the long legacy of hybridization in vascular plant evolution. Plant J 2023;114:743–66. 10.1111/tpj.16142 36775995

[CIT0075] Tadey M. Indirect effects of grazing intensity on pollinators and floral visitation. Ecol Entomol 2015;40:451–60. 10.1111/een.12209

[CIT0076] Tadey M. Reshaping phenology: livestock has stronger effects than climate on flowering and fruiting phenology in desert plants. Perspect Plant Ecol Evol Syst 2020;42:125501. 10.1016/j.ppees.2019.125501

[CIT0074] Tadey M, Tadey JC, Tadey N. Reproductive biology of five native plant species from the Monte desert of Argentina. Bot J Linn Soc 2009;161:190–201. 10.1111/j.1095-8339.2009.01001.x

[CIT0077] Vidal-Russell R, Tadey M, Urfusová R et al Evolutionary importance of the relationship between cytogeography and climate: new insights on creosote bushes from North and South America. Plant Divers 2022;44:492–8. 10.1016/j.pld.2021.11.006 36187552 PMC9512640

[CIT0082] Wells PV, Hunziker JH. Origin of the creosote bush (*Larrea*) deserts of southwestern North America. Ann Mo Bot Gard 1976;63:843–61. 10.2307/2395251

[CIT0083] White TJ, Bruns T, Lee S, et al Amplification and direct sequencing of fungal ribosomal RNA genes for phylogenetics. In: Gelfand MA, Sninsky JJ DH, White TJ (eds.), Pcr Protocols: A Guide to Methods and Applications. NY, USA: Academic Press, 1990. 315–22.

[CIT0084] Wu SD, Lin L, Li HL et al Evolution of Asian interior arid-zone biota: evidence from the diversification of Asian *Zygophyllum* (Zygophyllaceae). PLoS One 2015;10:e0138697. 10.1371/journal.pone.0138697 26393796 PMC4579068

[CIT0085] Wu SD, Zhang LJ, Lin L et al Insights into the historical assembly of global dryland floras: the diversification of Zygophyllaceae. BMC Evol Biol 2018;18:1–10. 10.1186/s12862-018-1277-z 30413147 PMC6234786

[CIT0078] Yang TW. Major chromosome races of *Larrea divaricata* in North America. J Ariz Acad Sci 1970;6:41–5. 10.2307/40022846

[CIT0079] Yang TW, Hunziker JH, Poggio L et al Hybridization between South American jarilla and North American diploid creosote bush (*Larrea*, Zygophyllaceae). Plant Syst Evol 1977;126:331–46.

[CIT0080] Yang TW, Yang YA, Xiong Z. Paternal inheritance of chloroplast DNA in interspecific hybrids in the genus *Larrea* (Zygophyllaceae). Am J Bot 2000;87:1452–8. 10.2307/2656871 11034920

[CIT0081] Yao H, Song J, Liu C et al Use of ITS2 region as the universal DNA barcode for plants and animals. PLoS One 2010;5:e13102. 10.1371/journal.pone.0013102 20957043 PMC2948509

[CIT0087] Zhou YF, Abbott RJ, Jiang ZY et al Gene flow and species delimitation: a case study of two pine species with overlapping distributions in southeast China. Evolution 2010;64:2342–52. 10.1111/j.1558-5646.2010.00988.x 20298431

[CIT0088] Zhou Y, Duvaux L, Ren G et al Importance of incomplete lineage sorting and introgression in the origin of shared genetic variation between two closely related pines with overlapping distributions. Heredity 2017;118:211–20. 10.1038/hdy.2016.72 27649619 PMC5315522

