# Supplementary material for: Everything everywhere all at once? Disentangling the long-lasting riddle of phylogenetic relationships and cryptic hybridization in the amphitropical genus Larrea
Source: AoB Plants. 2025 Apr 25;17(3):plaf024. doi: 10.1093/aobpla/plaf024 (PMC12190803; doi:10.1093/aobpla/plaf024)

Supporting Information Figures

Everything Everywhere All at Once? Disentangling the long-lasting riddle of phylogenetic relationships and cryptic hybridization in the amphitropical genus *Larrea*

Figure S1: **A** and **B** Schematic chromatograms of “Pure haplotypes” with no ambiguities at any base pair and with a particular combination of base-pairs for a given species; and **C** Schematic chromatograms of “Putative haplotypes” (PH), or sequences that showed double peaks, with two putative genetic bases per variable site, in this case with C/G overlap, for the analyses such sequence corresponding to one individual, was split into two putative haplotypes PH1-PH2. The arrow indicates double peaks with overlapped signals.

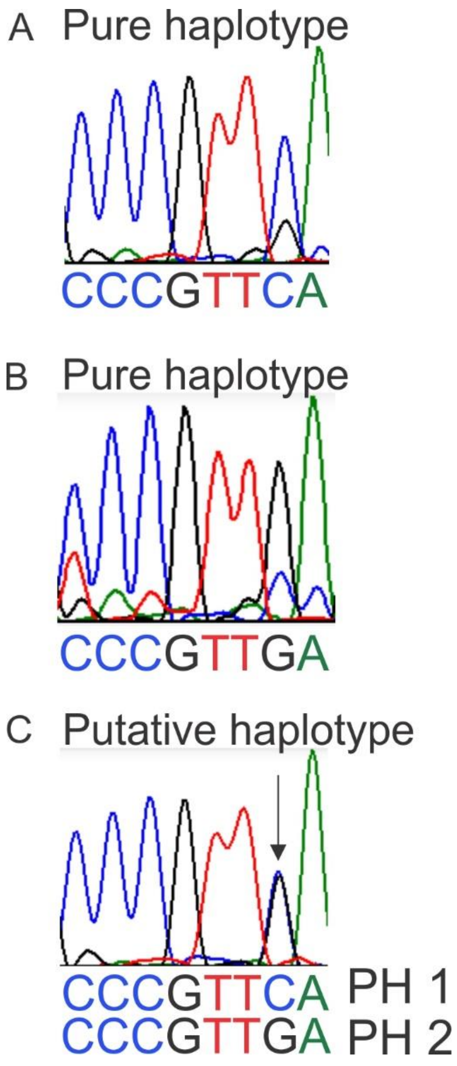

Figure S2: **A** Haplotype network using ITS. Circle size is proportional to haplotype frequencies and between-haplotype branch length is proportional to the number of mutational changes. Pure haplotypes are shown in **bold**. Putative haplotypes (PH1) are represented in lighter colors. **B** Phylogenetic relationships among five *Larrea* species for pure and PH1 ITS haplotypes. The above branch numbers represent Bayesian posterior probabilities, and branch length is proportional to the number of changes.

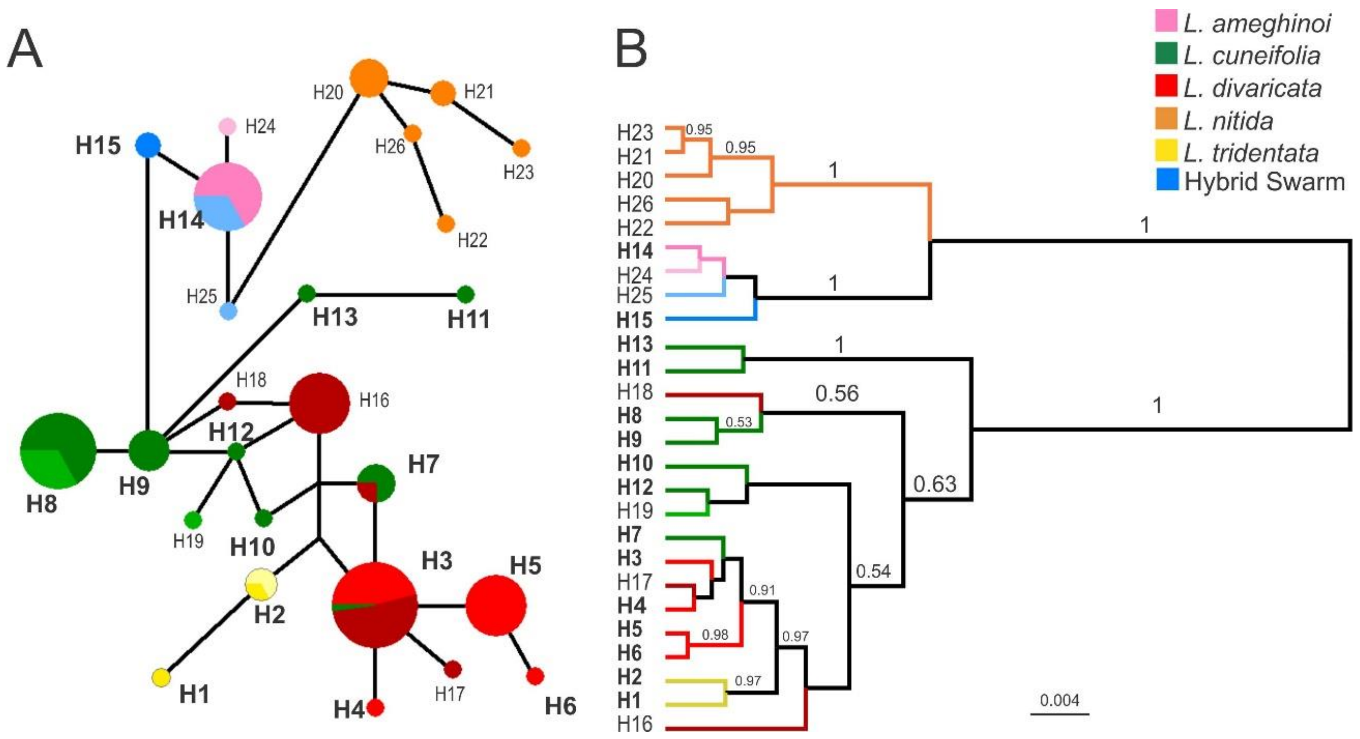

Pure haplotypes are in **bold**, and putative haplotypes (PH1) are represented in lighter colors. Colors in branches correspond to morpho-species. Color code: *La* in pink range, *Lc* in green range, *Ld* in red range, *Ln* in orange, *Lt* in yellow, HS in cyan range.

Figure S3: Complete phylogeny using ITS. Genera *Tribulus, Fagonia, Bulnesia, Guiaicum* and *Zygophyllum* were used as outgroups. Numbers above each branch indicate the posterior probability estimated via Bayesian inference using Beast 1.10.4. Numbers in the lower corner of each branch indicate estimated ages in Myr, using secondary calibration. Numbers in red and blue depict calibration points and estimated node ages, respectively.

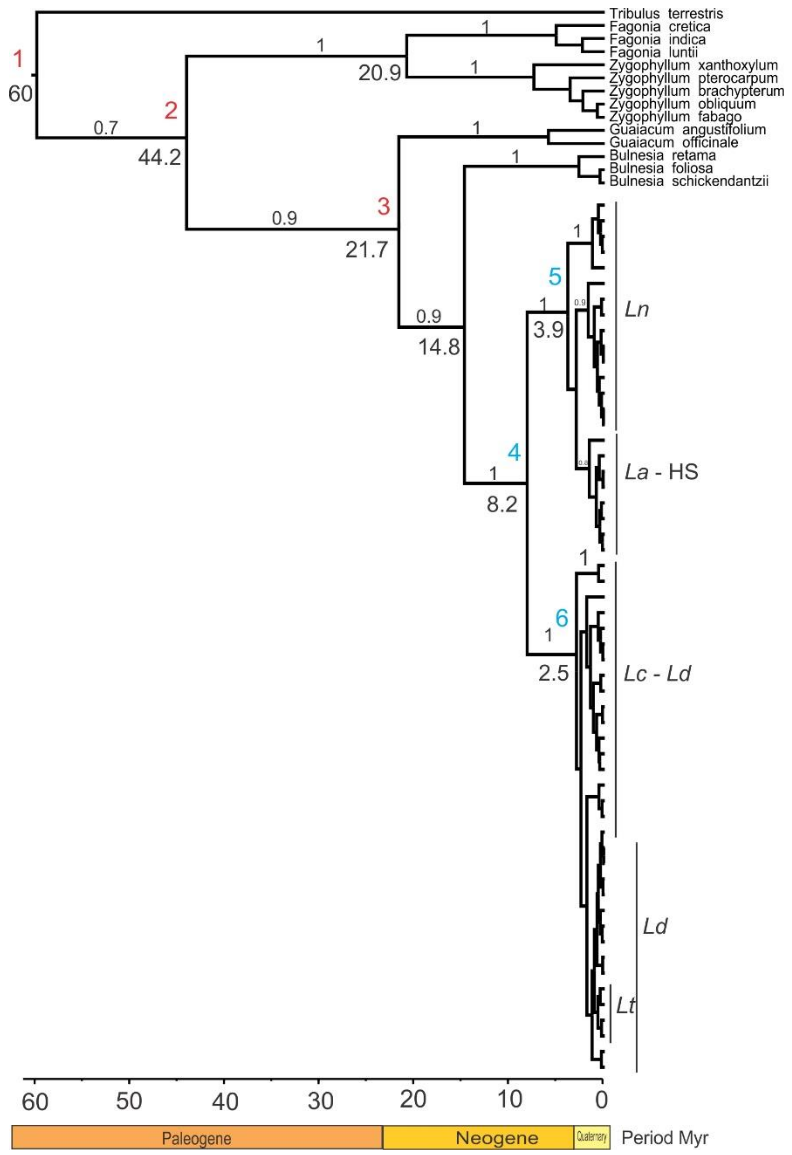

Figure S4: Complete phylogeny using rbcL chloroplast gene. Genera *Tribulus, Fagonia, Bulnesia, Guiaicum*, and *Zygophyllum* were used as outgroups. Numbers above each branch indicate the posterior probability estimated via Bayesian inference using Beast 1.10.4. Numbers in the lower corner of each branch indicate estimated ages in Myr, using secondary calibration. Numbers in red and blue depict calibration points and estimated node ages, respectively.

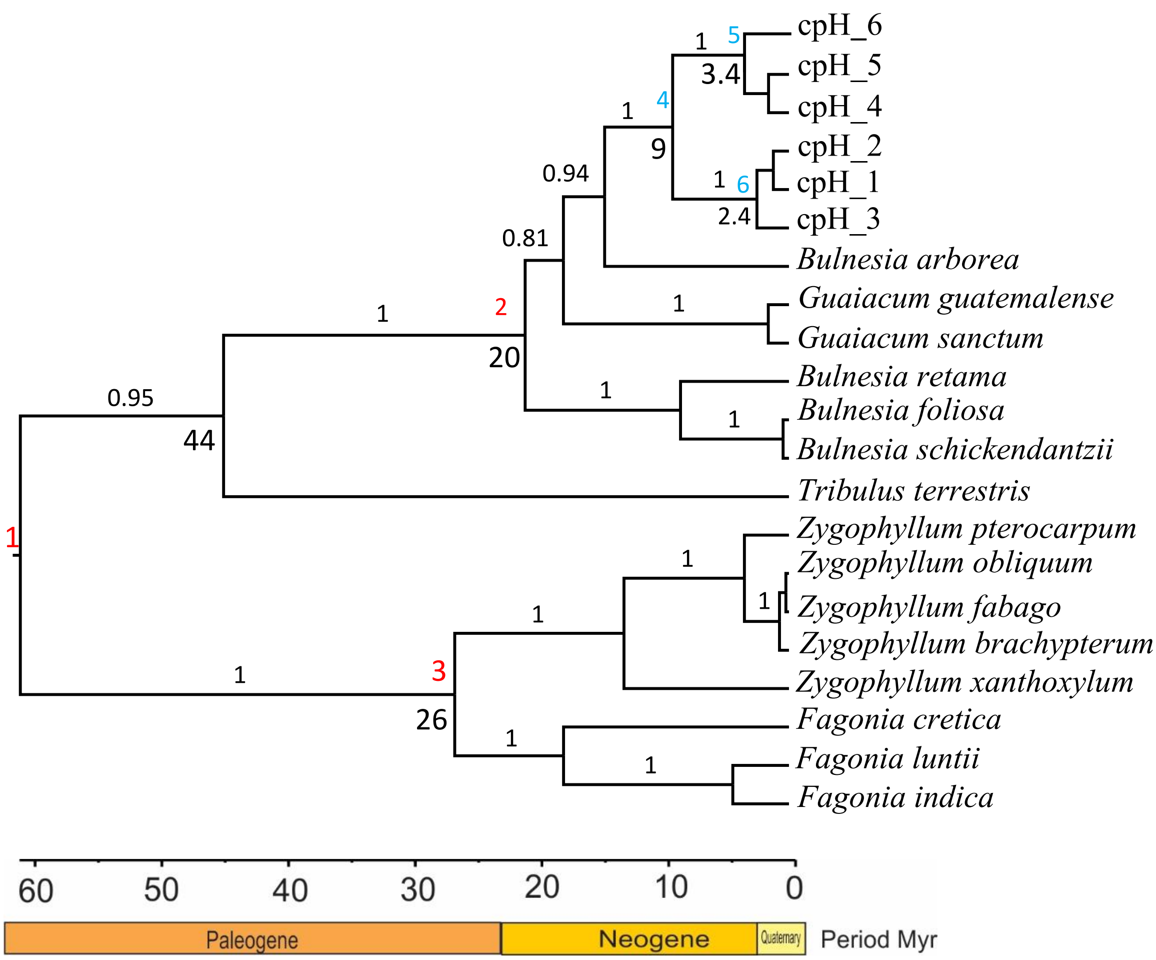

Supplement: plaf024_suppl_Supplementary_Figures [file plaf024_suppl_supplementary_figures.pdf]
