# Supplementary material for: Everything everywhere all at once? Disentangling the long-lasting riddle of phylogenetic relationships and cryptic hybridization in the amphitropical genus Larrea
Source: AoB Plants. 2025 Apr 25;17(3):plaf024. doi: 10.1093/aobpla/plaf024 (PMC12190803; doi:10.1093/aobpla/plaf024)
Supplement: plaf024_suppl_Supplementary_Tables [file plaf024_suppl_supplementary_tables.pdf]

Supporting Information Tables

Everything Everywhere All at Once? Disentangling the long-lasting riddle of phylogenetic relationships and cryptic hybridization in the amphitropical genus *Larrea*

Table S1: Summary of previous published phylogenetic studies in the genus *Larrea*, including: Sample size, DNA markers used in each work, details on phylogenetic analyses, and the corresponding reference.

Species (number of analysed individuals)	DNA markers	Phylogeny (with the caption from paper)	Reference
L. tridentata (40), *L. divaricata* (10), *L. cuneifolia* (9), *L. nitida* (6), *L. ameghinoi* (1) Outgroup *Bulnesia retamo*	Chloroplast rbcL and matK.	Parsimony tree. Ploidy levels, sample size and leaf shapes are also indicated. 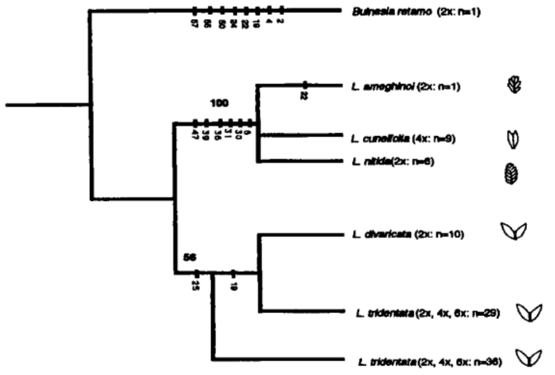	Hunter 1995
L. tridentata (1), *L. divaricata* (1), *L. cuneifolia* (1), *L. nitida* (1), *L. ameghinoi* (1) Outgroup: *P. chilensis; G. guatemalensis; B. arborea; P. tetracantha; F. indica; A. capensis; Z. simplex*	Chloroplast rbcL and nuclear ITS-1 and ITS-2	Combined parsimony tree 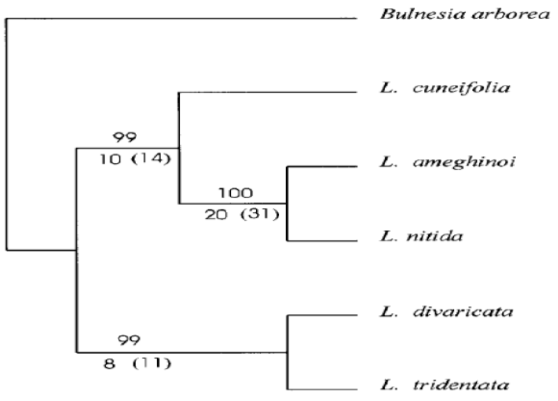	Lia et al., 2001

<table>
<tr>
<td>L. tridentata (92),
L. divaricata (1),
L. cuneifolia (1),
L. nitida (2).
Outgroup:
G. coulteri</td>
<td>Chloroplast psbA-trnH, rpl32-trnL, rpl16, rpoB-trnC, petN-trnC, and nuclear ITS</td>
<td>Consensus phylogeny: Chloroplast (left) and nuclear (right).
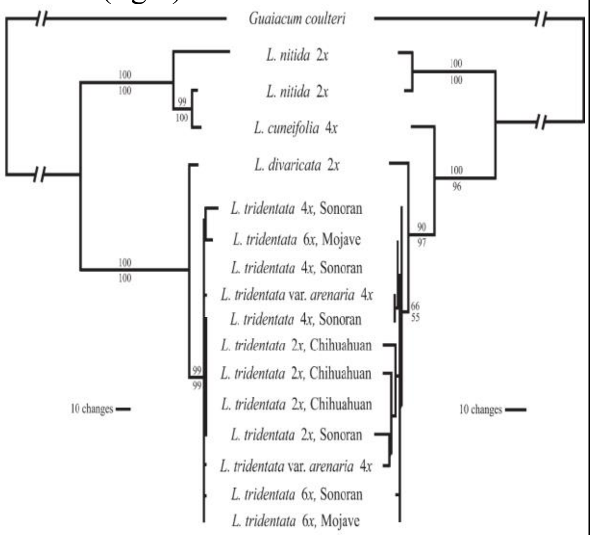
</td>
<td>Laport et al., 2012</td>
</tr>
</table>

Table S2: Detailed methods of PCR conditions

PCR master Mix	PCR condition	PCR products
10% trehalose 6.25 µl; ddH20 2 µl; 10X PCR buffer for Platinum Taq (Invitrogen) 1.25 µl; 50 mM MgCl2 (Invitrogen) 0.625 µl; 10 µM primer A (Invitrogen) 0.125 µl; 10 µM primer B (Invitrogen) 0.125 µl; 10 mM dNTPs mix (Kapa Biosystem) 0.0625 µl; Polymerase (5 U/µl) Platinum Taq polymerase –Invitrogen- or Phusion Hot Start High-Fidelity DNA Polymerase –Finnzymes-) 0.06 µl; DNA template 2 µl per well	For ITS2 (ITS-S2F/ITS4): 94°C for 5 min; 35 cycles of 94°C for 30 sec, 56°C for 30 sec, 72°C for 45 sec; final extension 72°C for 10 min.<br><br>For rbcL 94°C for 4 min; 35 cycles of 94°C for 30 sec, 55°C for 30 sec, 72°C for 1 min; final extension 72°C for 10 min.	PCR products were purified according to Werle et al. (1994) and sequenced at the CCDB in Canada or in Macrogen, Korea, using the BigDye Terminator Cycle Sequencing Kit v.3.1 (Thermo Scientific Inc.) on a 96-Well GeneAmp PCR System 9700 (Life Technologies, LT), with slightly modified PCR conditions: 0.4 µL BigDye Terminator v.3.1, 1 µL 3.2 µM primer, 1.8µL 5× sequencing buffer (LT), 2µL 1 M trehalose.

Table S3: Accession number of outgroup taxa for ITS and rbcL markers.

Outgroup	ITS	rbcL
Tribulus terrestris	AY260972	OM488276
Fagonia cretica	AY641624	MT645207
F. indica	AY641632	MW645246
F. luntii	AY641638	AJ133856
Zygophyllum xanthoxylum	DQ267180	KP087795
Z. pterocarpum	KU047981	JF944809
Z. brachypterum	KR002024	KU047970
Z. obliquum	KU047978	KU047978
Z. fabago	KR002029	MW368973
Guaiacum angustifolium	-	AY260974
G. officinale	-	MH432139
G. sanctum	-	MH549868
G. guatemalense	-	Y15019
Bulnesia retama	MH378456	MN525796
B. foliosa	MH378455	MH357632
B. schickendantzii	MH378457	H35763

Table S4: Age constraints used for normal prior distribution in the BEAST analyses including mean and standard deviation for different Zygophyllaceae nodes according to previous studies and molecular markers.

Crown Zygophyllaceae	Crown Zygophylloideae	Crown Larroideae	Markers	Reference
64 (sd= 14)	52.5 (sd= 11)	28.5 (sd= 10)	rbcL, trnL-trnF, trnS-trnG	Böhnert et al. 2020
58 (sd= 12)	54.2 (sd= 12)	23.3 (sd= 12)	rbcL	Wu et al. 2015
56.9 (sd= 22)	44.1 (sd= 20)	21.6 (sd= 14)	ITS	Wu et al. 2015
54.8 (sd= 16)	50.9 (sd= 12)	17.2 (sd= 12)	ITS, trnL, trnF	Wu et al. 2015

Table S5: Divergence time estimations for the Zygophyllaceae phylogeny using ITS nuclear marker. Calibration points and estimate divergence time in Myr. Node numbers correspond to those in Fig. S3. (Mean = node age, Min = minimum age, Max = maximum age).

Node	Calibration points (max-min)	Estimated divergence (max-min)
1. Crown Zygophyllaceae *Tribulus*	60 (94-30)	
2. Crown Zygophylloideae *Fagonia* and *Zygophyllum*	50 (67-41)	
3. Crown Larroideae *Guaiacum* / *Bulnesia*, *Larrea*	23 (29-12)	
4. *Larrera* diversifications		8.2 (13.5-5.4)
5. *L. nitida*/*L. ameghinoi*		3.9 (6.6-2.5)
6. *L. cuneifolia*, *L. divaricata* and *L. tridentata*		2.5 (5.1-1.9)

Table S6: Divergence time estimations for the Zygophyllaceae phylogeny using rbcL chloroplast gen. Calibration points used to estimate divergence time in Myr. Node numbers correspond to those in Fig. S4. (Mean = node age, Min = minimum age, Max = maximum age).

Node	Calibration points (max-min)	Estimated divergence (max-min)
1. Crown Zygophyllaceae *Tribulus*	60 (94-30)	
2. Crown Zygophylloideae *Fagonia* and *Zygophyllum*	50 (67-41)	
3. Crown Larroideae *Guaiacum* / *Bulnesia*, *Larrea*	23 (29-12)	
4. *Larrea* diversifications		9.08 (18-0)
5. cp Haplotypes 4, 5, 6		3.46 (8-0)
6. cp Haplotypes 1, 2, 3		2.49 (6-0)

Table S7: Sampled species´ individuals, population localities, haplotype (frequency), observed variation, herbarium voucher, and GeneBank accession numbers for ITS nuclear sequences of five *Larrea* species.

Species	Population acronym	Haplotype (frequency)	Variation	Pure/Putative haplotypes PH1 – PH2	Voucher specimen	Accession number
Lt	NM-SC	H1 (1)	Pure	H1	BCRUST103-007	PP989231
Lt	TC-SNP	H2 (3)	Pure	H2	BCRUST103-021	PP989232
Lt	NM-SC		PH1	H2 – H27	BCRUST103-008	
Lt	TC-SNP		PH1	H2 – H28	BCRUST103-022	
Lc	SA-CA	H3 (48)	Pure	H3	BCRUST33-048	PP989233
Ld	SA-LC		Pure	H3	BCRUST15-1054	
Ld	SA-LC		Pure	H3	BCRUST15-1052	
Ld	SA-LC		Pure	H3	BCRUST15-1053	
Ld	SA-LC		PH1	H3 – H5	BCRUST15-1050	
Ld	SA-CA		Pure	H3	BCRUST15-046	
Ld	SA-CA		PH1	H3 – H29	BCRUST15-1049	
Ld	CA-FBL		PH1	H3 – H31	BCRUST15-201	
Ld	CA-FBL		PH1	H3 – H32	BCRUST15-200	
Ld	LR-LB		Pure	H3	BCRUST15-87	
Ld	LR-LB		Pure	H3	BCRUST15-88	
Ld	LR-LB		Pure	H3	BCRUST15-89	
Ld	CD-LBM		PH1	H3 – H5	BCRUST15-58	
Ld	CD-LBM		PH1	H3 – H16	BCRUST15-55	
Ld	SJ-LC		Pure	H3	BCRUST15-71	
Ld	SJ-LC		PH1	H3 – H5	BCRUST15-73	
Ld	SJ-LP		PH1	H3 – H32	BCRUST15-70	
Ld	SJ-CA		Pure	H3	BCRUST15-016	
Ld	SJ-CA		Pure	H3	BCRUST15-117	
Ld	SL-JU		PH1	H3 – H54	BCRUST15-61	
Ld	ME-US		PH1	H3 – H32	BCRUST15-67	
Ld	ME-US		PH1	H3 – H32	BCRUST15-68	
Ld	ME-US		PH1	H3 – H32	BCRUST15-69	
Ld	SL-CQ		Pure	H3	BCRUST15-114	
Ld	SL-CQ		Pure	H3	BCRUST15-007	
Ld	ME-LC		PH1	H3 – H16	BCRUST15-64	
Ld	ME-LC		Pure	H3	BCRUST15-65	
Ld	ME-LC		Pure	H3	BCRUST15-66	
Ld	ME-GA		Pure	H3	BCRUST15-024	
Ld	ME-GA		Pure	H3	BCRUST15-125	
Ld	ME-GA		PH1	H3 – H34	BCRUST15-026	
Ld	ME-RG		Pure	H3	BCRUST15-83	
Ld	ME-RG		PH1	H3 – H16	BCRUST15-84	
Ld	ME-RG		PH1	H3 – H16	BCRUST15-85	
Ld	LP-LR		PH1	H3 – H16	BCRUST15-205	
Ld	LP-LR		PH1	H3 – H16	BCRUST15-63	
Ld	RN-CTN		Pure	H3	BCRUST15-98	
Ld	RN-CTN		Pure	H3	BCRUST15-62	
Ld	RN-CTN		PH1	H3 – H37	BCRUST15-97	

Ld	LP-LC		PH1	H3 – H5	BCRUST15-003	
Ld	NE-CH		PH1	H3 – H5	BCRUST15-034	
Ld	NE-CH		Pure	H3	BCRUST15-135	
Ld	RN-VA		PH1	H3 – H5	BCRUST15-76	
Ld	RN-VA		PH1	H3 – H5	BCRUST15-77	
Ld	RN-VA		PH1	H3 – H5	BCRUST15-78	
Ld	RN-LG		Pure	H3	BCRUST15-045	
Ld	RN-RN3		PH1	H3 – H5	BCRUST15-209	
Ld	RN-RN3		PH1	H3 – H35	BCRUST15-211	
Ld	SA-CA	H4 (1)	Pure	H4	BCRUST15-047	PP989234
Ld	SA-LC	H5 (11)	PH2	H3 – H5	BCRUST15-1050	PP989235
Ld	CD-LBM		PH2	H3 – H5	BCRUST15-58	
Ld	SJ-LC		PH2	H3 – H5	BCRUST15-73	
Ld	LP-ED		Pure	H5	BCRUST15-94	
Ld	LP-ED		Pure	H5	BCRUST15-95	
Ld	LP-LC		PH2	H3 – H5	BCRUST15-003	
Ld	NE-CH		PH2	H3 – H5	BCRUST15-034	
Ld	RN-VA		PH2	H3 – H5	BCRUST15-76	
Ld	RN-VA		PH2	H3 – H5	BCRUST15-77	
Ld	RN-VA		PH2	H3 – H5	BCRUST15-78	
Ld	RN-RN3		PH2	H3 – H5	BCRUST15-209	
Ld	RN-LG	H6 (1)	Pure	H6	BCRUST15-043	PP989236
Lc	ME-GA	H7 (4)	Pure	H7	BCRUST33-022	PP989237
Lc	LR-LA		Pure	H7	BCRUST33-045	
Lc	NE-CH		Pure	H7	BCRUST33-034	
Ld	LP-LC		PH1	H7 – H34	BCRUST15-104	
Lc	SJ-LP	H8 (15)	Pure	H8	BCRUST33-64	PP989238
Lc	SJ-LP		PH1	H8 – H38	BCRUST33-65	
Lc	SJ-LP		Pure	H8	BCRUST33-66	
Lc	ME-US		PH1	H8 – H9	BCRUST33-61	
Lc	SL-SQ		Pure	H8	BCRUST33-001	
Lc	ME-LC		Pure	H8	BCRUST33-58	
Lc	ME-LC		Pure	H8	BCRUST33-59	
Lc	ME-GA		Pure	H8	BCRUST33-023	
Lc	RN-CTN		PH1	H8 – H9	BCRUST33-89	
Lc	RN-VA		Pure	H8	BCRUST33-86	
Lc	RN-VA		Pure	H8	BCRUST33-87	
Lc	RN-VA		Pure	H8	BCRUST33-88	
Lc	RN-RN3		PH1	H8 – H38	BCRUST33-90	
Lc	RN-RN3		Pure	H8	BCRUST33-91	
Lc	RN-RN3		PH1	H8 – H9	BCRUST33-92	
Lc	ME-US	H9 (5)	PH2	H8 – H9	BCRUST33-61	PP989239
Lc	LR-LA		Pure	H9	BCRUST33-043	
Lc	SL-SQ		Pure	H9	BCRUST33-004	
Lc	RN-CTN		PH2	H8 – H9	BCRUST33-89	
Lc	RN-RN3		PH2	H8 – H9	BCRUST33-92	
Lc	SJ-CA	H10 (1)	Pure	H10	BCRUST33-012	PP989240

Lc	SJ-CA	H11 (1)	Pure	H11	BCRUST33-013	PP989241
Lc	LR-LA	H12 (1)	Pure	H12	BCRUST33-044	PP989242
Lc	NE-CH	H13 (1)	Pure	H13	BCRUST33-033	PP989243
La	RN-MA	H14 (12)	Pure	H14	BCRUST104-1	PP989244
La	RN-MA		Pure	H14	BCRUST104-2	
La	RN-RN3		Pure	H14	BCRUST104-7	
La	RN-RN3		Pure	H14	BCRUST104-8	
La	RN-RN3		Pure	H14	BCRUST104-9	
La	RN-RN3		Pure	H14	SISS8511	
La	RN-RN3		Pure	H14	SISS8510	
La	RN-RN3		Pure	H14	SISS854	
La	RN-RN3		PH1	H14 – H15	BCRUST105-4	
La	RN-RN3		PH1	H14 – H52	BCRUST105-6	
La	RN-RN3		PH1	H14 – H51	BCRUST105-9	
HS	RN-RN3		PH1	H14 – H50	BCRUST105-10	
HS	RN-RN3	H15 (2)	Pure	H15	BCRUST105-8	PP989245
HS	RN-RN3		PH2	H14 – H15	BCRUST105-4	
Ld	CA-FBL	H16 (9)	PH1	H16 – H33	BCRUST15-202	PP989246
Ld	CD-LBM		PH2	H3 – H16	BCRUST15-55	
Ld	CD-LBM		PH1	H16 – H56	BCRUST15-56	
Ld	ME-LC		PH2	H3 – H16	BCRUST15-64	
Ld	ME-RG		PH2	H3 – H16	BCRUST15-84	
Ld	ME-RG		PH2	H3 – H16	BCRUST15-85	
Ld	LP-LR		PH2	H3 – H16	BCRUST15-63	
Ld	LP-LR		PH2	H3 – H16	BCRUST15-205	
Ld	RN-SP		PH1	H16 – H36	BCRUST15-60	
Ld	SA-CA	H17 (1)	PH1	H17 – H30	BCRUST15-1048	PP989247
Ld	CD-LBM	H18 (1)	PH1	H18 – H55	BCRUST15-59	PP989248
Lc	SJ-CA	H19 (1)	PH1	H19 – H39	BCRUST33-11	PP989249
Ln	ME-US	H20 (4)	PH1	H20 – H41	BCRUST5-31	PP989250
Ln	ME-US		PH1	H20 – H40	BCRUST5-29	
Ln	RN-MA		PH1	H20 – H47	BCRUST5-34	
Ln	RN-RN3		PH1	H20 – H42	BCRUST5-38	
Ln	RN-MA	H21 (2)	PH1	H21 – H46	BCRUST5-33	PP989251
Ln	RN-RN3		PH1	H21 – H43	BCRUST5-39	
Ln	RN-MS	H22 (1)	PH1	H22 – H45	BCRUST5-32	PP989252
Ln	RN-LG	H23 (1)	PH1	H23 – H48	BCRUST5_010	PP989253
La	RN-MS	H24 (1)	PH1	H24 – H49	BCRUST104-3	PP989254
HS	RN-RN3	H25 (1)	PH1	H25 – H53	BCRUST105-1	PP989255
Ln	RN-SP	H26 (1)	PH1	H26 – H44	BCRUST5-290	PP989256
Lt	SC	H27 (1)	PH2	H2 – H27	BCRUST103-008	PP989257
Lt	TC	H28 (1)	PH2	H2 – H28	BCRUST103-022	PP989258
Ld	SA-CA	H29(1)	PH2	H3 – H29	BCRUST15-1049	PP989259
Ld	SA-CA	H30 (1)	PH2	H17 – H30	BCRUST15-1048	PP989260
Ld	CA-FBL	H31 (1)	PH2	H3 – H31	BCRUST15-201	PP989261
Ld	CA-FBL	H32 (6)	PH2	H3 – H32	BCRUST15-200	PP989262

Ld	SJ-LP		PH2	H3 – H32	BCRUST15-70	
Ld	ME-US		PH2	H3 – H32	BCRUST15-67	
Ld	ME-US		PH2	H3 – H32	BCRUST15-68	
Ld	ME-US		PH2	H3 – H32	BCRUST15-69	
Ld	RN-VA		PH2	H3 – H32	BCRUST15-77	
Ld	CA-FBL	H33 (1)	PH2	H16 – H33	BCRUST15-202	PP989263
Ld	ME-GA	H34 (2)	PH2	H3 – H34	BCRUST15-026	PP989264
Ld	LP-LC		PH2	H7– H34	BCRUST15-104	
Ld	RN-RN3	H35 (1)	PH2	H3 – H35	BCRUST15-211	PP989265
Ld	RN-SP	H36 (1)	PH2	H16 – H36	BCRUST15-60	PP989266
Ld	RN-CT	H37 (1)	PH2	H3 – H37	BCRUST15-97	PP989267
Lc	SJ-LP	H38 (2)	PH2	H8 – H38	BCRUST33-65	PP989268
Lc	RN-RN3		PH2	H8 – H38	BCRUST33-90	
Lc	SJ-CA	H39 (1)	PH2	H19 – H39	BCRUST33-11	PP989269
Ln	ME-US	H40 (1)	PH2	H20 – H40	BCRUST5-29	PP989270
Ln	ME-US	H41 (1)	PH2	H20 – H41	BCRUST5-31	PP989271
Ln	RN-RN3	H42 (1)	PH2	H20 – H42	BCRUST5-38	PP989272
Ln	RN-RN3	H43 (1)	PH2	H21 – H43	BCRUST5-39	PP989273
Ln	RN-SP	H44 (1)	PH2	H26 – H44	BCRUST5-290	PP989274
Ln	RN-MA	H45 (1)	PH2	H22 – H45	BCRUST5-32	PP989275
Ln	RN-MA	H46 (1)	PH2	H21 – H46	BCRUST5-33	PP989276
Ln	RN-MA	H47 (1)	PH2	H20 – H47	BCRUST5-34	PP989277
Ln	RN-LG	H48 (1)	PH2	H23 – H48	BCRUST5-010	PP989278
La	RN-MA	H49 (1)	PH2	H24 – H49	BCRUST104-3	PP989279
HS	RN-RN3	H50 (1)	PH2	H14 – H50	BCRUST105-10	PP989280
HS	RN-RN3	H51 (1)	PH2	H14 – H51	BCRUST105-9	PP989281
HS	RN-RN3	H52 (1)	PH2	H14 – H52	BCRUST105-6	PP989282
HS	RN-RN3	H53 (1)	PH2	H25 – H53	BCRUST105-1	PP989283
Ld	SL-DJ	H54 (1)	PH2	H3 – H54	BCRUST15-61	PP989284
Ld	CD-LBM	H55 (1)	PH2	H18 – H55	BCRUST15-59	PP989285
Ld	CD-LBM	H56 (1)	PH2	H16 – H56	BCRUST15-56	PP989286

Species: Sampled species: La: L. ameghinoi; Lc: L. cuneifolia; Ld: L. divaricata; Ln: L. nitida; Lt: L. tridentata; HS: Hybrid swarm.
Population acronym: Sampled localities (Province-locality). Population acronyms: NM-SC: New Mexico, Socorro County; TC-SNP: Tucson, Saguaro NP; SA-LC: Salta, Los Cardones; SA-CA: Salta, Cafayate; CA-FBL: Catamarca, Fiambalá; LR-LB: La Rioja, Laguna Brava; LR-FA: La Rioja, Famatina; LR-LA: La Rioja, La Rioja; SJ-SG: San Juan, San Guillermo; CD-LBM: Córdoba, Lucio Mansilla; SJ-LC: San Juan, La Ciénaga; SJ-LP: San Juan, Los Papagayos; SJ-CA: San Juan, Caucete; SL-JU: San Juan, Junín; ME-US: Mendoza, Uspallata; SL-SQ: San Luis, Sierra de las Quijadas; ME-LC: Mendoza, Lujan de Cuyo; ME-GA: Mendoza, General Alvear; ME-RG: Mendoza, Rio Grande; LP-ED: La Pampa, El Durazno; LP-LR: La Pampa, La Reforma; RN-CTN: Río Negro, Catriel Norte; RN-CTS: Río Negro, Catriel Sur; LP-LC: La Pampa, Lihuel Calel; NE-CH: Neuquén, El Chocón; RN-VA: Río Negro, Valcheta; RN-SC: Río Negro, Sierra Colorada; RN-SA: Río Negro, San Antonio; RN-LG: Río Negro, Las Grutas; RN-MA: Río Negro, Maquinchao; RN-RN3: Río Negro, Ruta Nacional 3; RN-SP: Río Negro, Sierra Paileman; CH-MA: Chubut, Madryn. See Table 1 for detailed geographic location.
Haplotype (frequency): Haplotype reference number used in the main document and figures including its frequency in brackets. See Table 1 for more details.
Variation: Pure = Sequence with no ambiguities at any base pair and with a particular combination of base-pairs for a given species; PH1 and PH2: Putative haplotypes = Sequences that showed double peaks, with two putative genetic bases per variable site.
Pure/PH1 and PH2: Number of haplotypes or haplotypes observed in each sampled individual.
Voucher specimen: Registered voucher of sampled individuals deposited in the BCRU (Centro Regional Universitario Bariloche) or SI (Darwinion) herbariums.
Accession number: Haplotype´ sequence accession code deposited in GenBank.

Table S8: Sampled individuals, species, localities, haplotype (frequency), observed variation, and GeneBank accession number for rbcL chloroplast sequences of five *Larrea* species.

Species	Population acronym	Haplotype rbcL (frequency)	Voucher specimen	Accession number
Lt	NM-SC	cpH_1 (4)	BCRUT103-008	PP947924
Lt	NM-SC		BCRUT103-007	
Lt	TC-SNP		BCRUT103-021	
Lt	TC-SNP		BCRUT103-022	
Lc	SJ-SG	cpH_2 (36)	BCRUST33-057	PP947925
Lc	SJ-SG		BCRUST33-050	
Ld	SA-LC		BCRUST15-1050	
Ld	SA-LC		BCRUST15-1052	
Ld	SA-LC		BCRUST15-1054	
Ld	SA-CA		BCRUST15-046	
Ld	SA-CA		BCRUST15-047	
Ld	SA-CA		BCRUST15-1048	
Ld	SA-CA		BCRUST15-1049	
Ld	CD-LBM		BCRUST15-58	
Ld	CD-LBM		BCRUST15-56	
Ld	CD-LBM		BCRUST15-55	
Ld	SJ-CA		BCRUST15-117	
Ld	SL-JU		BCRUST15-61	
Ld	ME-US		BCRUST15-67	
Ld	SL-CQ		BCRUST15-007	
Ld	SL-CQ		BCRUST15-114	
Ld	SL-CQ		BCRUST15-008	
Ld	SL-CQ		BCRUST15-013	
Ld	ME-LC		BCRUST15-65	
Ld	ME-GA		BCRUST15-026	
Ld	RN-CT		BCRUST15-62	
Ld	LP-LC		BCRUST15-001	
Ld	LP-LC		BCRUST15-002	
Ld	LP-LC		BCRUST15-003	
Ld	LP-LC		BCRUST15-104	
Ld	LP-LC		BCRUST15-004	
Ld	NE-CH		BCRUST15-033	
Ld	NE-CH		BCRUST15-034	
Ld	RN-LG		BCRUST15-043	
Ld	RN-LG		BCRUST15-044	
Ld	CH-MA		BCRUST15-048	
Ld	CH-MA		BCRUST15-049	
Ld	CH-MA		BCRUST15-050	
Ld	CH-MA		BCRUST15-051	
Ld	ME-GA	cpH_3 (2)	BCRUST15-024	PP947926
Ld	NE-CH		BCRUST15-036	
Lc	SA-CA	cpH_4 (36)	BCRUST33-046	PP947927
Lc	SA-CA		BCRUST33-049	

Lc	LR-LA		BCRUST33-042	
Lc	LR-LA		BCRUST33-043	
Lc	LR-LA		BCRUST33-044	
Lc	LR-FA		BCRURVR228	
Lc	SJ-LP		BCRUST33-66	
Lc	SJ-CA		BCRUST33-013	
Lc	SJ-CA		BCRUST33-001	
Lc	SL-SQ		BCRUST33-04b	
Lc	SL-SQ		BCRUST33-05	
Lc	ME-GA		BCRUST33-025	
Lc	ME-GA		BCRUST33-024	
Lc	NE-CH		BCRUST33-035	
Lc	RN-VA		BCRUST33-87	
Lc	RN-VA		BCRUST33-86	
Ln	LP-LC		BCRUST5-001	
Ln	LP-LC		BCRUST5-002	
Ln	LP-LC		BCRUST5-003	
Ln	LP-LC		BCRUST5-010	
Ln	NE-CH		BCRUST5-027	
Ln	NE-CH		BCRUST5-028	
Ln	RN-LG		BCRUST5-014	
Ln	RN-SR		BCRUST5-011	
Ln	RN-SR		BCRUST5-012	
Ln	RN-RN3		BCRUST5-39	
Ln	RN-RN3		BCRUST5-38	
Ln	RN-MA		BCRUST5-34	
Ln	RN-MA		BCRUST5-33	
Ln	RN-MA		BCRUST5-32	
Ln	CH-MA		BCRUST5-019	
Ln	CH-MA		BCRUST5-020	
Ln	CH-MA		BCRUST5-021	
Ln	CH-MA		BCRUST5-022	
Ln	CH-MA		BCRUST5-023	
Ln	RN-LG	cpH_5 (1)	BCRUST5-018	PP947928
La	RN-RN3	cpH_6 (6)	BCRUST104-7	PP947929
La	RN-MA		BCRUST104-3	
La	RN-MA		BCRUST104-2	
La	RN-MA		BCRUST104-1	
Ln	ME-US		BCRUST5-31	
Ln	ME-US		BCRUST5-29	

Species: Sampled species: *La*: *L. ameghinoi*; *Lc: L. cuneifolia; Ld: L. divaricata; Ln: L. nitida; Lt*: *L. tridentata*.
Population acronym: Sampled localities (Province-locality). Population acronyms: NM-SC: New Mexico, Socorro County; TC-SNP: Tucson, Saguaro NP; SA-LC: Salta, Los Cardones; SA-CA: Salta, Cafayate; CA-FBL: Catamarca, Fiambalá; LR-LB: La Rioja, Laguna Brava; LR-FA: La Rioja, Famatina; LR-LA: La Rioja, La Rioja; SJ-SG: San Juan, San Guillermo; CD-LBM: Córdoba, Lucio Mansilla; SJ-LC: San Juan, La Ciénaga; SJ-LP: San Juan, Los Papagayos; SJ-CA: San Juan, Caucete; SL-JU: San Juan, Junín; ME-US: Mendoza, Uspallata; SL-SQ: San Luis, Sierra de las Quijadas; ME-LC: Mendoza, Lujan de Cuyo; ME-GA: Mendoza, General Alvear; ME-RG: Mendoza, Rio Grande; LP-ED: La Pampa, El Durazno; LP-LR: La Pampa, La Reforma; RN-CTN: Río Negro, Catriel Norte; RN-CTS: Río Negro, Catriel Sur; LP-LC: La Pampa, Lihuel Calel; NE-CH: Neuquén, El Chocón; RN-VA: Río Negro, Valcheta; RN-SC: Río Negro, Sierra Colorada; RN-SA: Río Negro, San Antonio; RN-

LG: Río Negro, Las Grutas; RN-MA: Río Negro, Maquinchao; RN-RN3: Río Negro, Ruta Nacional 3; RN-SP: Río Negro, Sierra Paileman; CH-MA: Chubut, Madryn.
See Table 1 for detailed geographic location.
Haplotype (frequency): Haplotype reference number used in the main document and figures including its frequency in brackets. See Table 1 for more details.
Voucher: Registered voucher of sampled individuals deposited in the BCRU (Centro Regional Universitario Bariloche) herbarium.
Accession number: Haplotype´ sequence accession code deposited in GenBank.
